# From ^13^C-lignin to ^13^C-mycelium: *Agaricus bisporus* uses polymeric lignin as a carbon source

**DOI:** 10.1126/sciadv.adl3419

**Published:** 2024-04-19

**Authors:** Katharina Duran, Michael Kohlstedt, Gijs van Erven, Cynthia E. Klostermann, Antoine H. P. America, Edwin Bakx, Johan J. P. Baars, Antonie Gorissen, Ries de Visser, Ronald P. de Vries, Christoph Wittmann, Rob N. J. Comans, Thomas W. Kuyper, Mirjam A. Kabel

**Affiliations:** ^1^ Laboratory of Food Chemistry, Wageningen University & Research, Bornse Weilanden 9, 6708 WG Wageningen, Netherlands.; ^2^ Institute of Systems Biotechnology, Saarland University, Campus A 1.5, 66123 Saarbrücken, Germany.; ^3^ Wageningen Food and Biobased Research, Wageningen University & Research, Bornse Weilanden 9, 6708 WG Wageningen, Netherlands.; ^4^ Biobased Chemistry and Technology, Wageningen University & Research, Bornse Weilanden 9, 6708 WG Wageningen Netherlands.; ^5^ Bioscience, Wageningen University & Research, Droevendaalsesteeg 1, 6708 PB Wageningen, Netherlands.; ^6^ Plant Breeding, Wageningen University & Research, 6708 PB Wageningen, Netherlands.; ^7^ CNC Grondstoffen, Driekronenstraat 6, 6596 MA Milsbeek, Netherlands.; ^8^ IsoLife bv, Droevendaalsesteeg 1, 6708 PB Wageningen, Netherlands.; ^9^ Fungal Physiology, Westerdijk Fungal Biodiversity Institute & Fungal Molecular Physiology, Utrecht University, Uppsalalaan 8, 3584 CT Utrecht, Netherlands.; ^10^ Soil Chemistry and Chemical Soil Quality Group, Wageningen University & Research, Droevendaalsesteeg 3a, 6708 PB Wageningen, Netherlands.; ^11^ Soil Biology Group, Wageningen University & Research, Droevendaalsesteeg 3a, 6708 PB Wageningen, Netherlands.

## Abstract

Plant biomass conversion by saprotrophic fungi plays a pivotal role in terrestrial carbon (C) cycling. The general consensus is that fungi metabolize carbohydrates, while lignin is only degraded and mineralized to CO_2_. Recent research, however, demonstrated fungal conversion of ^13^C-monoaromatic compounds into proteinogenic amino acids. To unambiguously prove that polymeric lignin is not merely degraded, but also metabolized, carefully isolated ^13^C-labeled lignin served as substrate for *Agaricus bisporus*, the world’s most consumed mushroom. The fungus formed a dense mycelial network, secreted lignin-active enzymes, depolymerized, and removed lignin. With a lignin carbon use efficiency of 0.14 (g/g) and fungal biomass enrichment in ^13^C, we demonstrate that *A. bisporus* assimilated and further metabolized lignin when offered as C-source. Amino acids were high in ^13^C-enrichment, while fungal-derived carbohydrates, fatty acids, and ergosterol showed traces of ^13^C. These results hint at lignin conversion via aromatic ring-cleaved intermediates to central metabolites, underlining lignin’s metabolic value for fungi.

## Teaser

*Agaricus bisporus* converts ^13^C-lignin into fungal protein, underlining lignin’s metabolic value for fungi.

## INTRODUCTION

Microbial conversion of plant biomass is essential for carbon cycling and sequestration (*1*, *2*). Plant biomass is mainly composed of densely intertwined cell wall (hemi-)cellulosic polysaccharides and the aromatic polymer lignin (*3*). The general consensus is that fungi depolymerize plant polysaccharides to monosaccharides, by using their extensive extracellular carbohydrate-degrading enzyme machineries. Monosaccharides are subsequently used in various fungal metabolic routes. Specific fungi, mainly Basidiomycota, can also degrade and mineralize lignin, while this aromatic polymer remains largely unaltered by many other (micro)organisms (*4*, *5*). Lignin’s recalcitrance is related to its heterogeneous and complex aromatic structure, providing the plant with mechanical strength and protection against microbial attack, and shields plant cell wall polysaccharides from degradation (*6*). Assimilation of lignin, and subsequent metabolization by fungi, stands against the long-standing paradigm that lignin is not used for growth and/or net energy gain (*7*–*9*). Contrary to this long-standing theory, a recent study has shown that two white-rot fungi, *Gelatoporia subvermispora* and *Trametes versicolor* (Basidiomycota; Polyporales), assimilated and converted 4-hydroxybenzoic acid and vanillic acid into proteinogenic amino acids (*10*). While elegantly demonstrating that such lignin-derived monoaromatic compounds can be used for fungal biomass generation, obviously, the uptake of freely accessible monomeric compounds is not the same as degrading and using (native) lignin. Hence, this raised the intriguing question whether not only such monomeric aromatic compounds but also native polymeric lignin can serve as carbon source for fungal biomass formation.

The edible white button mushroom, *Agaricus bisporus* (Basidiomycota, Agaricales), has been cultivated since the 18th century on recalcitrant waste streams that are largely composed of lignocellulose (*11*). During its mycelial growth, *A. bisporus* secretes a variety of oxidoreductases, underlying the substantial lignin oxidation, degradation, and delignification [40% (w/w)] observed (*12*–*15*). Moreover, it was demonstrated that *A. bisporus* mineralized ^14^C-labeled (homo-)polymeric aromatic model compounds to ^14^CO_2_ under axenic compost conditions (*16*). It is well accepted that *A. bisporus*’ ability to remove lignin during mycelial growth facilitates subsequent release of polysaccharides, which are used for fruiting body formation (*17*). We here pose the challenging hypothesis that substantial delignification is not exclusively driven by the benefits through access to polysaccharides but that lignin degradation products enter intracellularly and are used in *A. bisporus*’ central carbon metabolism. Besides being paramount for understanding fungal physiology, this would open up a myriad of possibilities for biotechnological valorization of polymeric lignin via fungal conversion.

Until now, no fungal growth study has been conducted using polymeric, uniformly ^13^C-labeled lignin as carbon source nor have corresponding ^13^C-enrichments from lignin or lignin degradation products been investigated in other fungal biomass compounds than amino acids, such as hexoses, ribose, fatty acids, and steroids. This gap in literature is likely a consequence of experimental and analytical challenges encountered when studying insoluble ^13^C-lignin rather than soluble ^13^C-aromatic monomers. To unambiguously attribute incorporated ^13^C in fungal biomass to lignin, the substrate has to be the sole carbon source and hence pure. Besides being pure, this lignin should be structurally comparable to that encountered in the natural habitat, i.e., contain substructures the fungus can act on, and thus should not be extensively converted/modified. These combined prerequisites make the lignin isolation challenging, especially when dealing with small amounts of valuable ^13^C-labeled plant biomass. Consequently, fungal treatment setups need to be adjusted and scaled down accordingly. Next, determination of (^13^C-) lignin yields upon fungal treatment is inherently challenging due to the low amounts of modified remaining (in)soluble lignin and interfering fungal biomass. Furthermore, insoluble residual lignin partially embedded in mycelium hampers determination of fungal biomass yield and consequently overestimates carbon use efficiency (CUE). Therefore, we corrected the fungal biomass yield at the end of the treatment by subtracting the initial fungal inoculum mass and the embedded lignin and were able to determine the real de novo formed fungal biomass when cultivated on lignin. Hence, we expressed the CUE as ratio of de novo formed fungal biomass over total ^13^C-lignin provided at the start of the treatment.

**Fig. 1.**
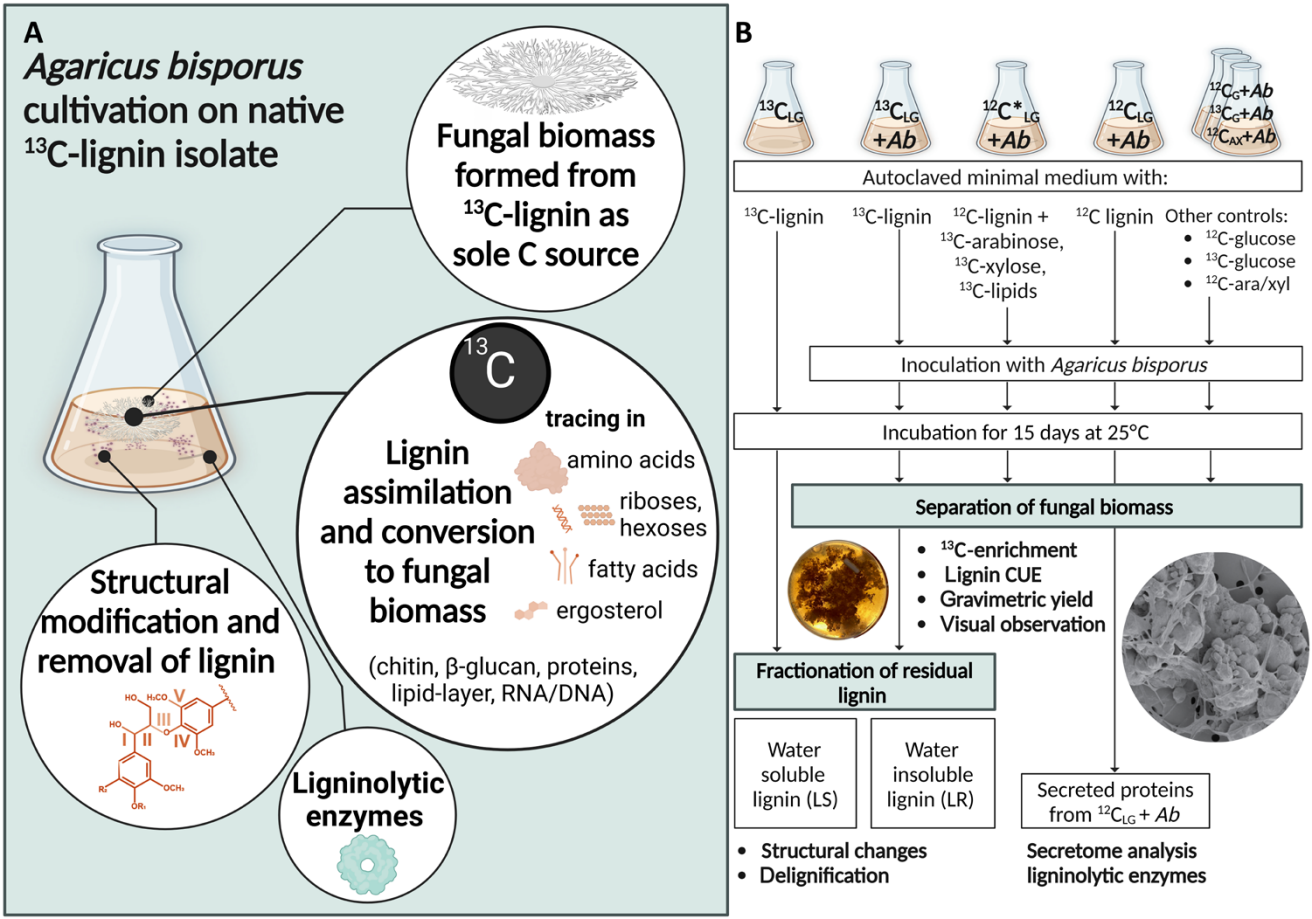
Overview of approach to assess whether *A. bisporus* structurally modifies and delignifies, assimilates and metabolizes ^13^C-isotope labeled lignin. Main research aims (**A**). Experimental setup and sample codes of lignin treatments by *A. bisporus* (**B**). C, carbon; LG, lignin; G, glucose; AX, arabinose and xylose mix [50/50 (w/w %)]; *Ab*, *A. bisporus*; CUE, carbon use efficiency.

In this study, these challenges have been carefully and thoroughly addressed. First, structurally intact ^13^C-labeled wheat straw lignin isolate was obtained by mild extraction, from uniformly labeled ^13^C-wheat straw (>97.7 atom % ^13^C; IsoLife), and purified to contain very low amounts of carbohydrate and lipid impurities. Quantifying residual ^13^C-lignin in (in)soluble and fungal mycelium fractions was possible on a <100 μg scale, by relying on the unique analytical pyrolysis–gas chromatography–mass spectrometry (GC-MS) infrastructure, here using a nonlabeled (^12^C) lignin isolate as internal standard (*18*). With these data, delignification, fungal biomass yield, and subsequently, CUEs were established. Last, controls with identically isolated nonlabeled lignin spiked with ^13^C-labeled monosaccharides and lipids in concentrations as present in the ^13^C-lignin isolate were conducted to establish that ^13^C-fractional enrichment of fungal biomass cultivated on ^13^C-lignin exceeded values of this control treatment (Fig. 1). Submerged fungal cultivations allowed to map structural changes of soluble and insoluble residual lignin products by heteronuclear single-quantum coherence (HSQC) nuclear magnetic resonance (NMR) and pyrolysis–GC-MS.

In short, we investigated whether *A. bisporus* forms fungal biomass from ^13^C-lignin, resulting in ^13^C-enriched fungal cell wall polysaccharides (i.e., β-glucan, glucose; chitin, glucosamine), ^13^C-enriched proteins (amino acids), ^13^C-enriched DNA and RNA (riboses), ^13^C-enriched lipids (fatty acids), and ^13^C-enriched steroids (ergosterol). To underpin lignin conversion into fungal biomass, (remaining) partially degraded lignin structural features were analyzed by ^1^H-^13^C HSQC NMR and pyrolysis–GC-MS, and *A. bisporus*’ secretomes were analyzed by proteomics. This study helps to deepen the understanding of fungal lignin conversion and contributes to redefining the concept of lignin carbon use by saprotrophic fungi.

## RESULTS

### From lignin to fungal biomass

In this experimental setup, *A. bisporus* was cultivated in duplicate for 15 days on pure native ^13^C-wheat straw lignin (table S1) as carbon source in liquid minimal medium (Fig. 1B; coded ^13^C_LG_ + *Ab*). Despite all isolation and purification efforts undertaken, reaching a purity of 93% (w/w), the final ^13^C-lignin isolate still contained (traces) of carbohydrates and lipids. Contaminants were mainly arabinose [3.5% (w/w)] and xylose [1.4% (w/w)], with traces of glucose [0.8% (w/w)], lipids [0.5% (w/w)], and traces of nitrogen (table S1). Therefore, serving as control treatment, *A. bisporus* was cultivated in duplicate with an identical nonlabeled lignin isolate spiked with ^13^C-labeled arabinose, ^13^C-xylose, ^13^C-glucose, and ^13^C-lipids (for composition, see table S2) in concentrations as present in the ^13^C-lignin isolate (Fig. 1B; coded ^12^C*_LG_ + *Ab*).

**Fig. 2.**
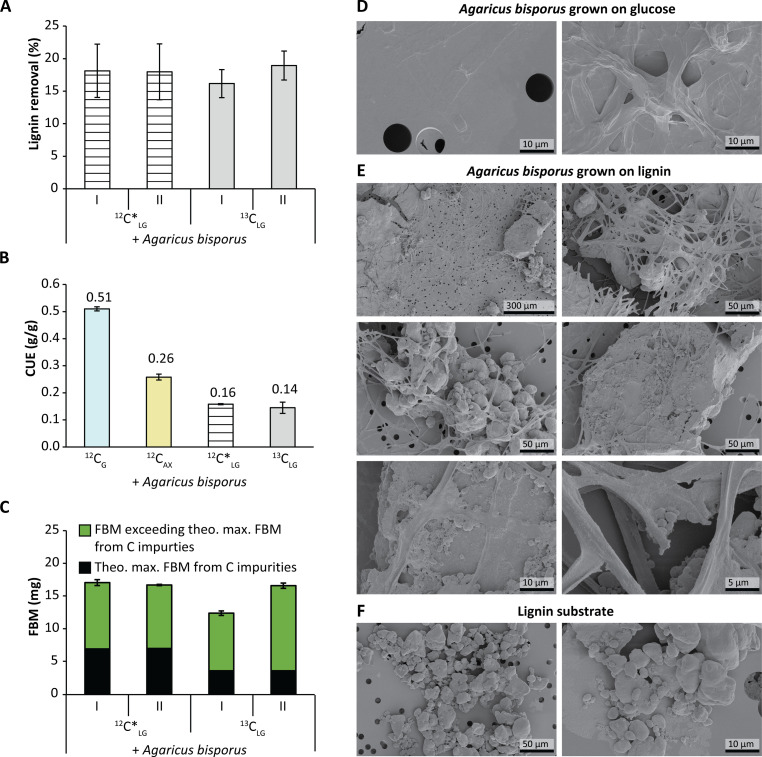
*A. bisporus* metabolizes lignin for fungal biomass formation. Removal of lignin by *A. bisporus* grown on nonlabeled lignin spiked with ^13^C-carbohydrates and lipids (^12^C*_LG_, striped bars) and ^13^C-labeled lignin (^13^C_LG_, gray bars). Delignification was calculated by mass recoveries of residual insoluble (LR), soluble (LS), and fungal biomass (FBM) fractions and lignin contents thereof analyzed by quantitative pyrolysis–GC-MS. Lignin removal was corrected for sample-handling losses, based on recoveries of uninoculated lignin controls (**A**). FBM per substrate (CUE); g/g) of *A. bisporus* cultivated on glucose (^12^C_G_, blue bar), arabinose-xylose [50:50 (w/w)] (^12^C_AX_, yellow bar), nonlabeled lignin spiked with ^13^C-carbohydrates and lipids (^12^C*_LG_, striped bar), and ^13^C-lignin (^13^C_LG_, gray bar). Values have been corrected for inoculation seed and lignin embedded in FBM (**B**). FBM formed (mg) extrapolated to 100 mg of substrate (C-source). Theoretically possible FBM (mg) based on C-containing impurities in the lignin isolate (black bar) (using a CUE of 0.5) and total measured FBM (mg, green plus black bars) generated on lignin media (**C**). Scanning electron microscopy (SEM) pictures of *A. bisporus* grown on glucose (**D**), grown on lignin (**E**), and of untreated lignin (uninoculated) (**F**).

In all treatments, the fungus properly developed (fig. S1) and formed a dense mycelial network thoroughly intertwined with remaining lignin (Fig. 2E). Absolute lignin removal was calculated by determining absolute mass and lignin contents of soluble (LS), insoluble (LR), and fungal biomass fractions, and corrected for losses occurring in the control lignin flask that was not inoculated with *A. bisporus* (e.g., losses due to sample handling). Absolute lignin removal was 18.1 and 17.6% (±0.4) (w/w) of lignin in ^12^C*_LG_ + *Ab* and ^13^C_LG_ + *Ab*, respectively (Fig. 2A).

To further understand whether this lignin decrease contributed to fungal growth, CUEs were calculated (Fig. 2B). Lignin CUEs were determined, which were 0.14 and 0.16 (±0.02), in ^13^C_LG_ + *Ab* and ^12^C*_LG_ + *Ab*, respectively (Fig. 2B). Initial fungal-growth experiments with divergent lignin sources resulted in similar lignin CUEs (table S3). As indicated above, determining lignin CUEs is not straightforward, which we addressed by distinguishing between de novo formed fungal biomass and co-collected intertwined remaining insoluble lignin.

Lignin-related CUEs have not been published for fungi so far, to the best of our knowledge. Only Rodriguez *et al*. (*19*) mentioned a conversion of lignin-like substrate into fungal biomass, but the setup of their research is not comparable with ours. To be specific, these authors used a mixture of lignin-derived monomers and oligomers following base-catalyzed depolymerization, a monocellular yeast (*Rhodotorula mucilaginosa*), and optical density measurements to determine growth yields. Furthermore, their CUEs were calculated using the biomass at the maximum growth rate, with substrate consumed (*19*).

One could argue that because the lignin isolates still contained low amounts of other C-containing impurities, this might consequently have led to overestimation of the lignin CUE (Fig. 2C). However, even when the theoretical amount of mycelium possibly formed from these impurities was calculated very conservatively (i.e., based on glucose CUE of 0.5), the total formed fungal biomass amounts well exceeded this value by 139 and 147% in ^12^C*_LG_ + *Ab* and by 248 and 367% in ^13^C_LG_ + *Ab* (Fig. 2C). As expected, CUEs for most impurities were lower, as already indicated for the arabinose-xylose mix, and carbohydrates were not completely consumed, thus further supporting that lignin was used as main carbon source by the fungus to form biomass.

Not only gravimetric and chemical analysis confirmed lignin conversion by *A. bisporus*, we also visually observed that the fungus properly developed. The mycelial network could be well distinguished from lignin by scanning electron microscopy (SEM; Fig. 2, E and F). The mycelium interacted and strongly entangled the lignin substrate. These visualizations could indicate that lignin conversion likely took place in proximity to the mycelium (fig. S2).

### *A. bisporus* caused *s*tructural modification of lignin

**Fig. 3.**
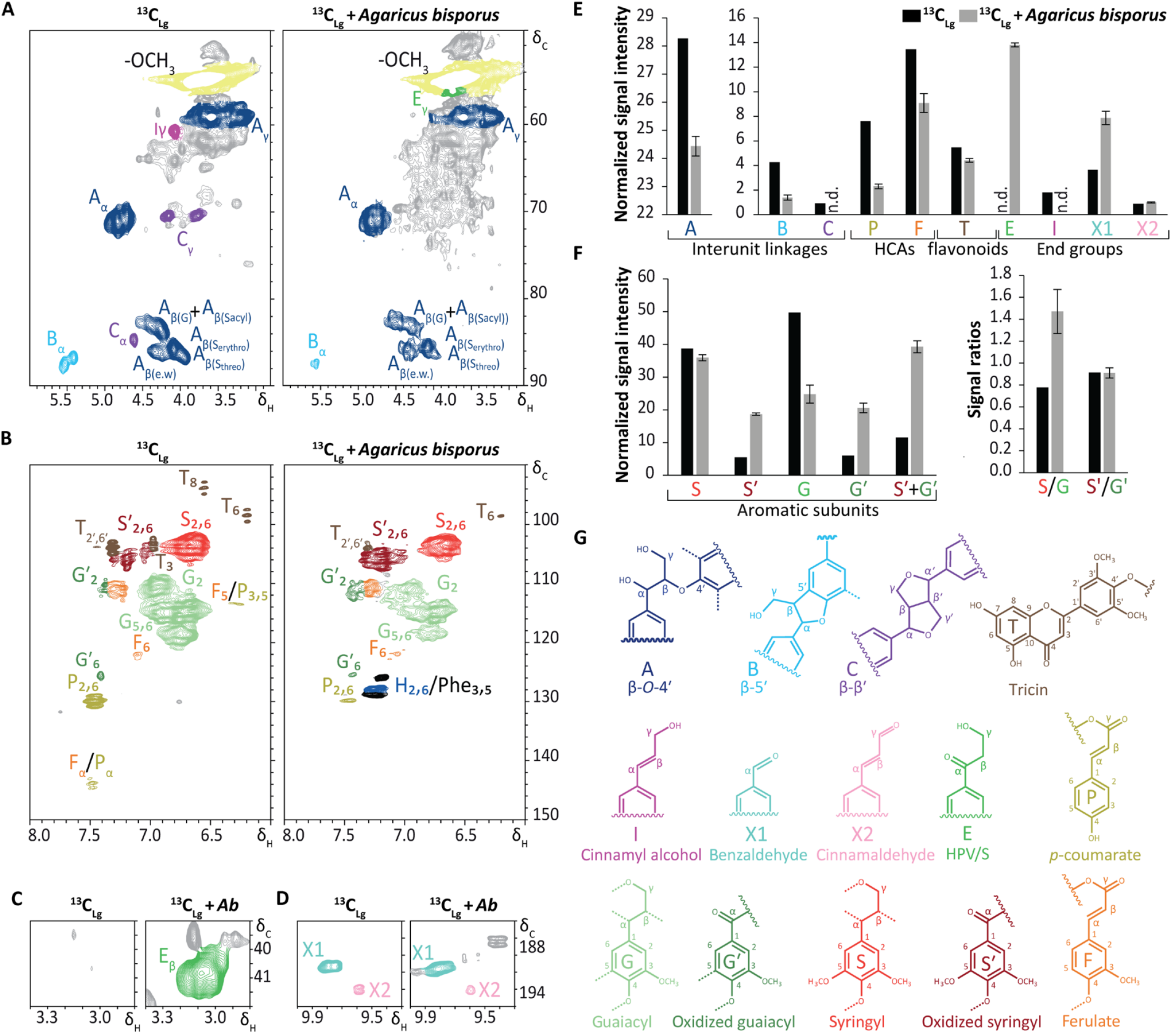
^1^H-^13^C HSQC NMR spectra of fungal treated lignin and controls from soluble lignin fraction (LS). Aliphatic regions (**A**), aromatic regions (**B**), region of HPV/S (**C**), and aldehyde regions (**D**) of ^1^H-^13^C HSQC NMR spectra of SPE-purified supernatant of untreated ^13^C-lignin control (LS^13^C_Lg_) and of *A. bisporus*–treated ^13^C-lignin (S^13^C_Lg_ + *Ab*) (right). Semiquantitative analysis of volume integrals based on normalized signal intensity of interunit linkages, hydroxycinnamic acids, flavonoids and end groups (**E**), and aromatic subunits and signal ratios (**F**) of SPE-purified ^13^C-lignin supernatants (black bars, LS^13^C_Lg_; gray bars, LS^13^C_Lg_ + *Ab*). Averages, including SDs, of LS^13^C_Lg_ + *Ab* are of biological duplicates. Color codes in the spectra correspond to structures in (**G**), and gray represents unassigned spectra. See Fig. 1 for explanation of sample codes.

Coinciding with delignification and fungal biomass formation when *A. bisporus* was cultivated on ^13^C-lignin, the remaining lignin substructures were severely structurally modified, indicating lignin cleavage and oxidation and therefore, extracellular conversion. Especially the soluble fractions (LS) accumulated conversion products, as evidenced by both pyrolysis–GC-MS (tables S4 to S7) and HSQC NMR (Fig. 3, table S6, and figs. S3 and S4). More specifically, both methods demonstrated an accumulation of Cα-oxidized substructures and diagnostic cleavage products at the expense of intact interunit linkages. The structural changes observed by HSQC NMR (Fig. 3 and table S7) matched those following from the characterization of lignin populations when *A. bisporus* was grown in an industrial composting environment (*15*), in terms of subunit preference (Fig. 3F) and the depletion of intact β-*O*-4-interunit linkages, hydroxycinnamates, and tricin (Fig. 3E). Likewise, similar diagnostic lignin cleavage products, especially benzaldehyde and hydroxypropiovanillone/syringone substructures, accumulated (Fig. 3, C and E), which can respectively be attributed to Cα-Cβ and β-*O* cleavage of β-*O*-4 aryl ethers. This suggests that despite the axenic lab-scale growth conditions applied here, *A. bisporus* relied on similar lignin modification strategies as compared with its industrial habitat. Somewhat unexpectedly, the lignin fraction that was densely entangled by mycelium (Fig. 2 and table S6) showed minimal structural alterations. Remaining insoluble lignin (LR) (tables S4 and S6) was apparently not as affected by fungal lignin modification and showed less structural changes compared with the soluble lignin fraction (LS). Nonetheless, the amount of residual lignin (LR fraction) was pronouncedly lower for the fungal-treated lignin compared with the untreated one (table S4).

### *A. bisporus* secretes lignin active enzymes

**Fig. 4.**
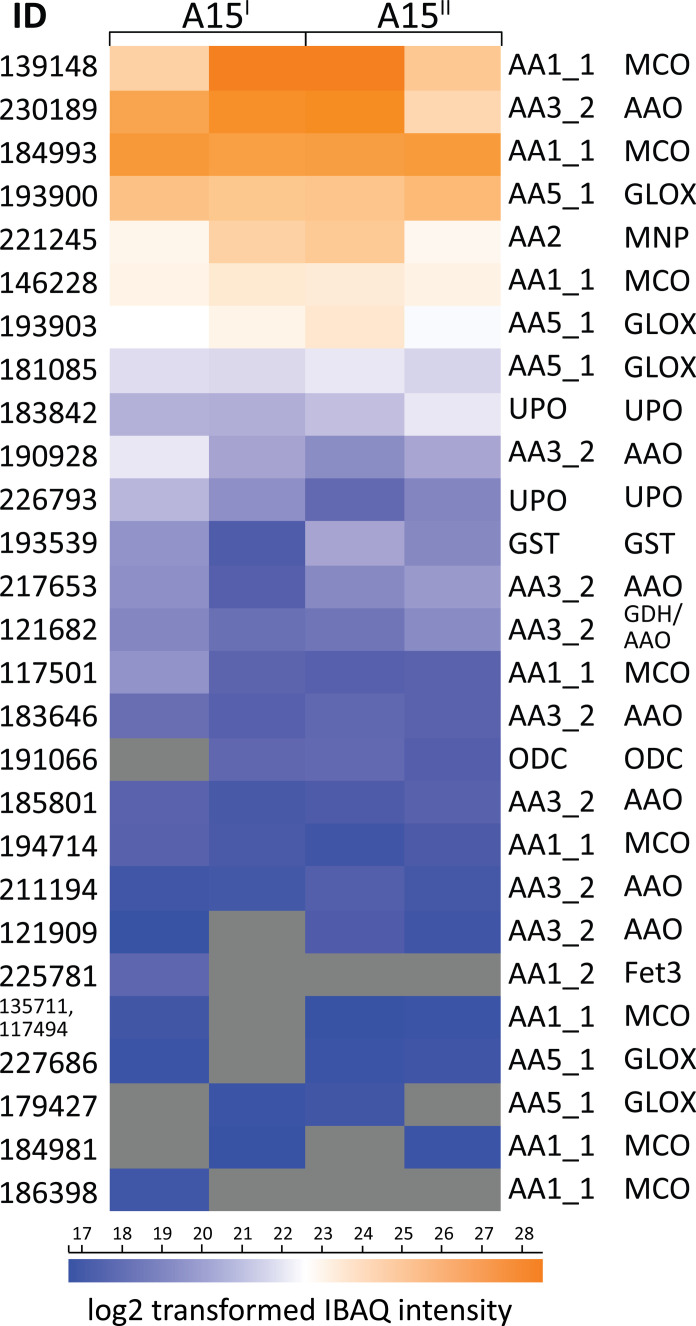
Secretome profiling of *A. bisporus* lignin treatments. Protein IDs and log2-transformed IBAQ intensities of enzymes active on lignin and aromatics in secretomes of biological duplicates (coded A15^I^ and A15^II^) in analytical duplicates of *A. bisporus* cultivated on nonlabeled lignin in minimal medium (secretomes of ^12^CLg + *Ab*; data S1). CAZyme family and subfamily, protein ID, and putative functions are provided if available [according to Joint Genome Institute identifier “jgi|Agabi_varbisH97_2” and Morin *et al.* (*11*)]. MCO, multicopper oxidase; UPO, unspecific peroxygenase; ODC, oxalate decarboxylase; MnP, manganese peroxidase; GLOX, glyoxal oxidase; AAO, aryl alcohol oxidase; GDH, glucose dehydrogenase; GST, glutathione *S*-transferase.

To extracellularly degrade lignin, fungi secrete a plethora of enzymes, mostly oxidoreductases under aerobic conditions. In the *A. bisporu*s–treated lignin (^12^C_Lg_ + *Ab*) secretome (data S1), a total of 500 (putative) proteins were detected, of which 72 were carbohydrate-active enzymes (CAZymes). Furthermore, we found 27 enzymes that are active on lignin and aromatics (Fig. 4 and data S1). Among those enzymes, 23 are classified as Auxiliary Activities (AA; www.CAZy.org) (*20*), of which eight belong to subfamily AA1_1, another eight to subfamily AA3_2, five to subfamily AA5_1, and one to subfamilies AA1_2 and AA2 (Fig. 4). Six AA1_1 members were annotated as sensu stricto laccases. Among the most abundant laccases, one enzyme (ID 139148; Fig. 4) was also pronouncedly secreted in *A. bisporus* secretomes obtained during the industrial-scale mycelium growth phase on a straw-based substrate, once more pinpointing to similar delignification strategies (*21*). Proteins from subfamilies AA5_1 and AA3_2 (Fig. 4) can be further categorized as glyoxal oxidases (GLOX) and aryl alcohol oxidases (AAO), respectively. These enzymes are likely involved in lignin modification and potentially supply peroxidases and peroxygenases with required H_2_O_2_ (*22*). Among the CAZy-classified proteins, one manganese peroxidase (MnP) (AA2; ID 221245; Fig. 4) was found, which likely is involved in ligninolysis (*23*). Two heme-containing unspecific peroxygenases (UPOs; ID 226793, 183842; Fig. 4), were also secreted, and as previously suggested (*21*, *24*) they may play a role in ligninolytic processes, either by demethylation, hydroxylation (*25*), or potentially by contributing to the cleavage of β-*O*-4′ ether linkages (*26*, *27*).

### Fungal biomass components enriched in ^13^C

**Fig. 5.**
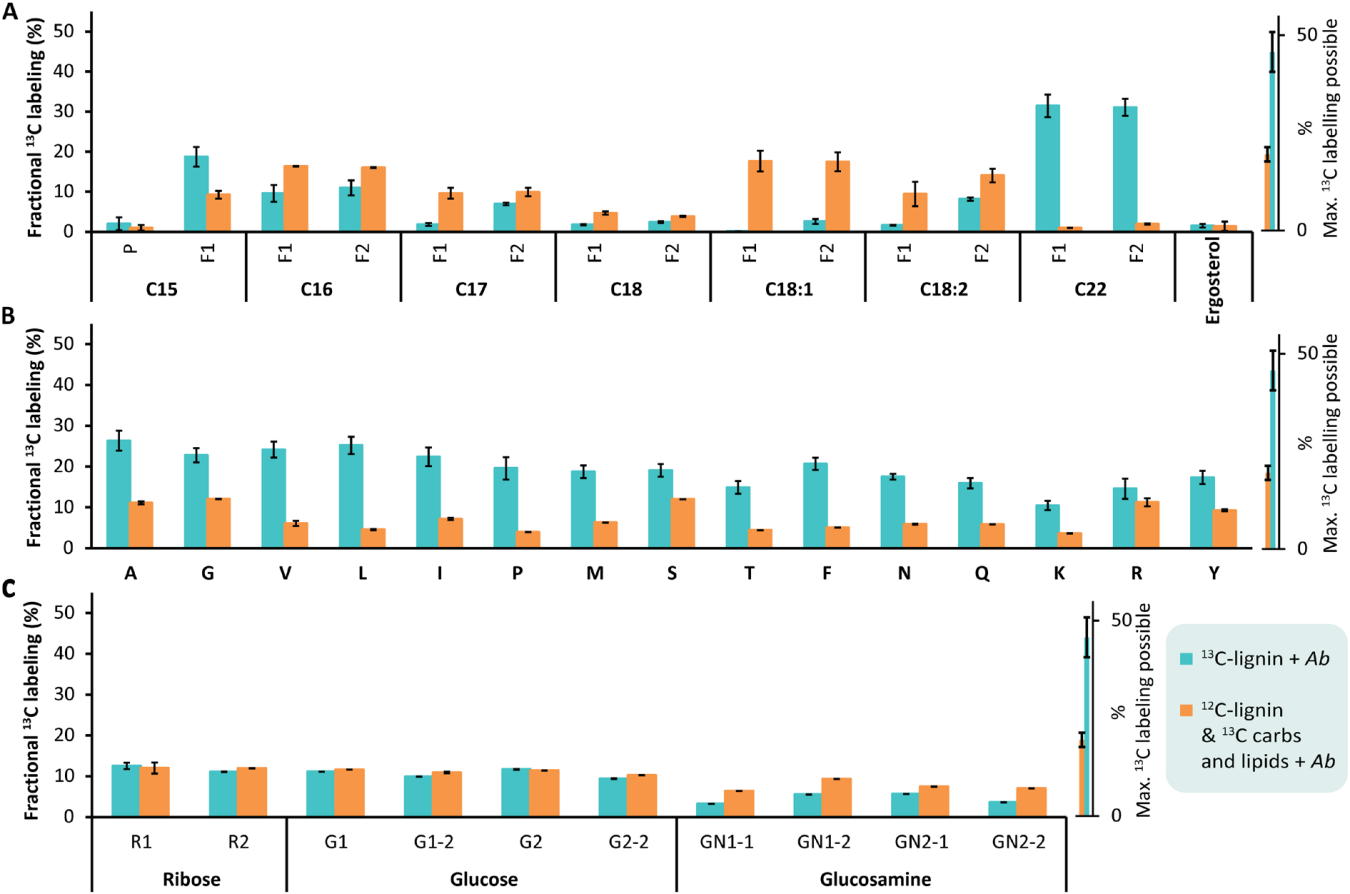
^13^C-fractional labeling of FBM compounds. Fatty acids and ergosterol (**A**), amino acids (**B**), carbohydrates (**C**), of *A. bisporus* (*Ab*) FBM from ^12^C*_Lg_ + *Ab* (orange bars; ^12^C-lignin “spiked” used as carbon source), and from ^13^C_Lg_ + *Ab* (turquoise bars; ^13^C-lignin used as carbon source). All results are the average of biological duplicates, and error bars represent the SDs. The graphs on the right side indicate maximal labeling possible, calculated from FBM increase and mycelium seed used, for ^13^C_Lg_ + *Ab* (turquoise), and labeled spike used for ^12^C*_Lg_ + *Ab* (orange).

Indisputable evidence for the *A. bisporus*–mediated conversion of (degraded) lignin to fungal biomass can only be obtained by tracing ^13^C-isotopes derived from lignin in *A. bisporus* biomass. Therefore, ^13^C-fractional enrichment was established for fungal marker molecules for protein (i.e., in amino acids), cell wall β-glucan (i.e., in hexoses), RNA and DNA (i.e., in ribosyl units of nucleic acids), lipids (i.e., in fatty acids), and ergosterol (Fig. 5). Outcomes were benchmarked against the control growth experiment of *A. bisporus* in which we spiked nonlabeled lignin with (traces of) ^13^C-isotopic arabinose, xylose, glucose, and lipids (^12^C*_Lg_), in amounts present as in ^13^C_Lg_ + *Ab*. In addition, *A. bisporus* was cultivated on ^13^C-labeled glucose, on nonlabeled glucose, and on nonlabeled lignin, to assess (absence of) ^13^C-isotope fractional enrichment. The unlabeled lignin control verified that lignin degradation products did not interfere with the ^13^C-enrichment analysis (fig. S7).

Note that, on the basis of the amount of mycelium inoculum used (nonlabeled) and the amount of de novo formed fungal biomass (labeled) during the treatment, the theoretical maximum of ^13^C-enrichment is 45.7 ± 5.1% for ^13^C_LG_ + *Ab*, respectively. For ^12^C*_Lg_ + *Ab* treatment, the maximum labeling was calculated based on the added amount of ^13^C-labeled monosaccharides and lipids, which was 19.5 ± 0.7% (Fig. 5). Fractional labeling was analyzed and calculated based on established methodology (fig. S8).

As hypothesized, in the treatment with *A. bisporus* cultivated on ^13^C-lignin, all fungal biomass components were ^13^C-isotope enriched, although the level of enrichment differed among the various components (Fig. 5). For fungal biomass harvested from ^13^C_LG_ + *Ab*, the ^13^C-fractional labeling was lowest (3.25 to 12.52%) for ribose, glucose, and glucosamine, which are building blocks of RNA and DNA, respectively, and of the cell wall polymers glucan and chitin. Similar (low) enrichments in ribose and glucose were reached for fungal biomass from ^12^C*_LG_ + *Ab*, signifying that ^13^C-enrichment in ^13^C_Lg_ + *Ab* in RNA/DNA and glucan might preferentially have resulted from the ^13^C-carbohydrates and/or fatty acids (i.e., spiked and/or present as impurity in the ^13^C-lignin isolate) rather than from the (converted) aromatics derived from ^13^C-lignin. For glucosamine, the ^13^C-enrichment was even higher for fungal biomass from ^12^C*_LG_ + *Ab* (6.4 to 9.3%; Fig. 5B) compared with that from ^13^C_LG_ + *Ab* (3.3 to 5.7%; Fig. 5B), hinting at an even lower preference and/or likelihood to convert lignin-substrate into chitin-like compounds. It can, nonetheless, be proposed that to a certain extent, ^13^C from lignin still ended up in fungal ribose, glucose, and glucosamine, since the mentioned impurities were bound or adsorbed to lignin, as they remained after thorough cleanup steps, hence becoming less freely available for the fungus in the ^13^C-lignin treatments as compared to the spiked control treatment. This suggestion is strengthened by the fact that only 74.0 ± 0.7% and 68.2 ± 11.1% of all carbohydrate impurities were metabolized from the treatment in ^13^C_Lg_ + *Ab* and ^12^C*_Lg_ + *Ab*, respectively.

The ^13^C-enrichments in the various fatty acid were low for fungal material of ^12^C*_Lg_ + *Ab* (1.0 to 17.7%; Fig. 5A). An exception was the long-chain fatty acid behenic acid (C22:0), where ^13^C_Lg_ + *Ab* reached 31.5% enrichment, compared with 2.0% in ^12^C*_Lg_ + *Ab*. Apparently, when *A. bisporus* was cultivated on lignin, C22:0 was biosynthesized more abundantly, or shorter fatty acids were elongated (i.e., from the fungal inoculum). Coherently, on the basis of fatty acid composition and content analysis (table S8), *A. bisporus* produced more C22:0 when cultivated on lignin compared with cultivation on glucose. Ergosterol, a steroid often used as measure for mycelium formation, was lower in ^13^C-fractional enrichment compared with the fatty acids and only reached 1.5 and 1.4%, in ^13^C_Lg_ + *Ab* and ^12^C*_Lg_ + *Ab*, respectively (Fig. 5A).

High ^13^C-enrichments were obtained for proteinogenic amino acids in the fungal biomass harvested from ^13^C_Lg_ + *Ab*, and ^13^C-enrichment in ^13^C_Lg_ + *Ab* (10.5 to 26.3%) exceeded that from ^12^C*_Lg_ + *Ab* (3.6 to 12%; Fig. 5C). Thus, this observation provides clear evidence that truly lignin-derived aromatics, and not solely impurities, were used for protein biosynthesis by *A. bisporus*. The highest ^13^C-fractional enrichment in fungal biomass from ^13^C_LG_ + *Ab* was reached in alanine (A, 26.3%), leucine (L, 25.2%), and valine (V, 24.1%), followed by glycine (G, 22.7%), isoleucine (I, 22.4%), phenylalanine (F, 20.7%), and proline (P, 19.6%; Fig. 5C).

Overall, ^13^C-fractional enrichment of fungal biomass components showed a clear trend that lignin was mostly used for amino acid formation. Still, ^13^C from lignin degradation products could have also been used for the biosynthesis of other (less ^13^C-enriched) fungal compounds (e.g., lipid, RNA, glucan, and chitin), rather than that these enrichments were solely the result of the ^13^C-impurities from the lignin isolate, especially as not all carbohydrate impurities from the substrates were removed.

## DISCUSSION

Contrary to long-standing theories that for fungi, the metabolic value of carbon derived from lignin is (close to) zero (*7*) and that lignin is only degraded or modified to allow access to energy-rich carbohydrates (*17*), recent studies have demonstrated that assimilation and conversion of monomeric aromatics into fungal amino acids occur (*10*). In this study, we established that carbon derived from polymeric lignin was used for *A. bisporus* biomass formation, rather than lignin being solely mineralized to CO_2_ extracellularly (*16*).

**Fig. 6.**
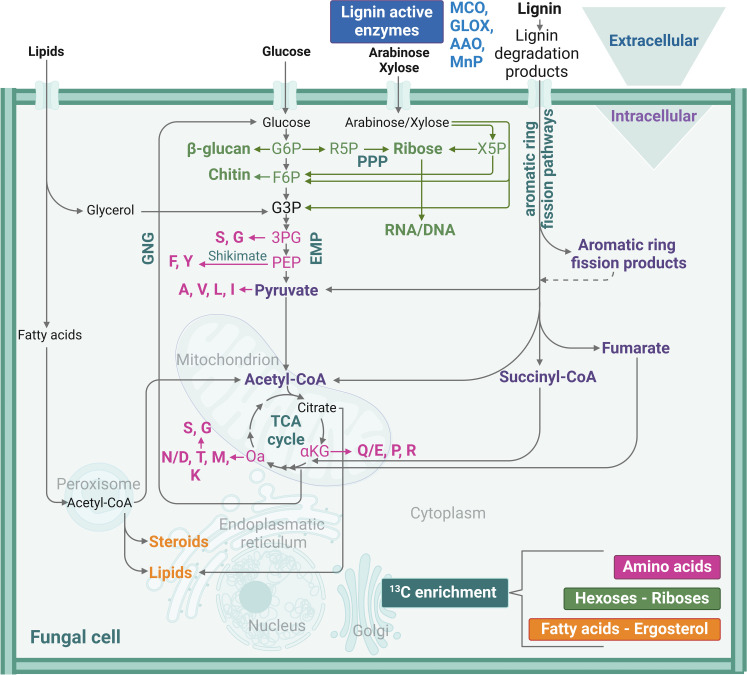
Extracellular lignin active enzymes and intracellular pathways and conversion of assimilated lipids, arabinose/xylose, glucose, and lignin degradation products in a fungal cell. Metabolic pathways show key intermediates where they branch off for biosynthesis of amino acids, hexoses/riboses, and lipids. Amino acids are shown in their one-letter codes (magenta). Key-branching points for biosynthesis of riboses, β-glucan, and chitin are glucose-6-phosphate (G6P) and fructose-6-phosphate (F6P; green). These fungal components/fibers can be hydrolyzed and result in hexoses and riboses (green) for determining the ^13^C-enrichment. Lipids and steroids are marked in yellow and ^13^C-fractional enrichment was tested in fatty acids and ergosterol. Extracellularly, lignin is depolymerized by lignin active enzymes (dark blue), which have been investigated in *A. bisporus* secretome cultivated on nonlabeled lignin. MCO, multicopper oxidase; PPP, pentose phosphate pathway; EMP, Embden-Meyerhof-Parnas pathway/glycolysis; GNG, gluconeogenesis; TCA cycle, tricarboxylic acid cycle; G3P, glycerol-3-phosphate; 3PG, 3-phosphoglycerate; PEP, phosphoenolpyruvate; X5P, xylose-5-phosphate; R5P, ribose-5-phosphate; αKG, α-ketoglutarate; Oa, oxaloacetate; PW, pathway. The figure was created with BioRender (BioRender.com). Metabolic pathways were based on the Kyoto Encyclopedia of Genes and Genomes (KEGG) database (*33*, *34*), and proposed end products of aromatic-ring cleavage pathways are based on the schemes proposed by Holesova *et al.* (*31*), Lubbers *et al*. (*29*), del Cerro *et al.* (*10*), Kijpornyongpan *et al.* (*5*), and Patyshakuliyeva *et al.* (*47*).

In this study, we differentiated between extracellular (i.e., lignin degradation) and intracellular (i.e., further conversion of degraded lignin) pathways (Fig. 6). In the fungal delignification study investigated here, structural modification of lignin and secretion of ligninolytic enzymes are indicative for the extracellular lignin modification by *A. bisporus*. Intracellular lignin conversion by the fungus was proven by ^13^C-fractional enrichment fungal biomass. Here, with lignin as carbon source and suboptimal growth conditions, the delignification was not as extensive as in optimized *A. bisporus* industrial solid-state growth conditions on wheat straw–based substrate, reaching up to 40% delignification (*13*, *15*). Nonetheless, the ~18% delignification reached in this study is substantial, and largely the same (oxidative) enzymes were identified in the secretome as previously found under optimal growth conditions (*24*). Whether the suboptimal conditions applied here hindered delignification (e.g., monoculture compared to usual compost-based cultivation, lignin as carbon source, submerged growth, depletion of minerals, absence of cofactors, etc.) or that certain residual lignin-structures were less susceptible for further degradation has yet to be researched.

*A. bisporus* further processed degraded (^13^C-) lignin intracellularly (Fig. 6) into fungal biomass components, as compellingly established here via comprehensive ^13^C tracing analysis when ^13^C-lignin was used as carbon source (fig. S8). With these results, the long-standing theory that *A. bisporus* only removes and structurally alters lignin to liberate carbohydrates is hereby disproven (*17*, *28*). Mostly, (depolymerized) ^13^C-lignin was converted into de novo synthesis of ^13^C-isotopic enriched proteinogenic amino acids in *A. bisporus* biomass, pointing at a metabolic preference to use carbon from depolymerized lignin for protein synthesis instead for de novo formation of cell wall polysaccharides (e.g., glucan and chitin) and of DNA/RNA. In this study, only fungal-bound derived amino acids were analyzed, while secreted extracellular proteins were not included in the ^13^C-enrichment analysis. Hence, we anticipate that the actual amount of ^13^C-labeled enzymes is even higher.

Currently, intracellular metabolic pathways of homocyclic aromatic compounds are not yet fully resolved for higher fungi. However, suggestions for fungal aromatic intracellular pathways have been published, and similarities with bacterial routes have been indicated (*29*). Del Cerro *et al*. (*10*) suggested that aromatic-ring cleavage products are funneled into intracellular pathways, although not all fungal (intermediate) metabolic enzymes have been identified (*10*, *29*). End products are suggested to be acetyl–coenzyme A (CoA) and succinyl-CoA, which are common central intermediates (*30*). In addition, others have described that the intermediates pyruvate and fumarate can result from ring-cleaved para-diphenolic benzene structures; for example, the monoaromatic compound 4-hydroxybenzoate is converted to gentisate before ring fission (*31*, *32*), although again not all corresponding enzymes have been identified. The above-suggested lower aromatic metabolic pathways, and in particular the four specified key intermediates, form the basis of the in Fig. 6 proposed *A. bisporus*’ metabolic pathways, indicating de novo synthesis routes of biomass components from lignin.

De novo synthesis of ^13^C-isotopic enriched alanine (A), leucine (L), isoleucine (I), and valine (V) reached highest ^13^C-enrichment levels when *A. bisporus* was grown on ^13^C-lignin (24.1 to 26.3%; ^13^C_Lg_ + *Ab*), indicating that pyruvate, which is a direct precursor for these amino acids (*33*, *34*), could be a key intermediate (Fig. 6). Concurrently, del Cerro *et al.* (*10*) found a similar high ^13^C-enrichment for alanine in *T. versicolor* grown on ^13^C-monoaromatic compounds. Conversion of acetyl-CoA to pyruvate is kinetically unfavorable and has not been reported, as it generally proceeds vice versa, catalyzed by pyruvate dehydrogenase (*35*). The aromatic amino acids tyrosine (Y) and phenylalanine (F) were ^13^C-enriched for 17.3 and 20.7%, respectively, in mycelium formed from ^13^C-lignin (^13^C_Lg_ + *Ab*). The ^13^C-enrichments of tyrosine and phenylalanine were lower compared with alanine, leucine, isoleucine, and valine and that could relate to the less direct pathway for de novo synthesis. Their synthesis (F, Y) from (ring-cleaved) aromatic compounds would first require gluconeogenesis (GNG) and glycolysis to generate the intermediate phosphoenolpyruvate (PEP; Fig. 6), which is a precursor of these amino acids. Thus, it can be anticipated that the biosynthesis of tyrosine and phenylalanine via the suggested multiple pathways is less favorable compared with the more direct formation of the intermediate pyruvate and subsequent synthesis of alanine, leucine, isoleucine, and valine (Fig. 6). A similar explanation could underlie the lower ^13^C-enrichment of the other amino acids, compared with A, L, I, and V, which result from intermediates of the tricarboxylic acid cycle (TCA) cycle such as oxaloacetate [Oa; lysine (K), threonine (T), methionine (M), and aspartate (N)], or from α-ketoglutarate [αKG; proline (P), arginine (R), and glutamate (Q) (Fig. 6)]. Serine (S) and glycine (G) can derive either form from L-threonine (T; by L-threonine aldolase to G and glycine hydroxymethyltransferase to S), or via the intermediate glycerol-3-phosphate (3PG) from the Embden-Meyerhof-Parnas (EMP) pathway. All these amino acids had lower ^13^C-enrichment ranging between 10.5 and 19.6% (^13^C_Lg_ + *Ab*) compared with A, L, I, and V. *A. bisporus* grown on ^13^C-glucose resulted in much higher enrichments of all amino acids (ranging between 39.4 and 61.4%, respectively; ^13^C_G_ + *Ab*; fig. S7). Glucose enters glycolysis directly (i.e., EMP pathway) and fuels the TCA cycle with acetyl-CoA (Fig. 6). Glucose is a more favorable substrate compared with lignin for *A. bisporus* to promote biosynthesis of amino acids.

Concurrently, de novo synthesis of β-glucan and chitin is also anticipated to first form central intermediates, e.g., pyruvate and acetyl-CoA (Fig. 6), from aromatic (ring-fission) products, which will further convert via GNG and EMP to glucose-6-phosphate (i.e., β-glucan), and further to fructose-6-phosphate (i.e., chitin). Again, this multitude of enzyme-catalyzed steps, hence metabolically unfavorable, might explain the rather low ^13^C- enrichment of chitin and glucan when based on ^13^C-lignin. Consistently, ^13^C-enrichments of chitin and glucan were higher in fungal biomass cultivated on ^13^C-glucose (^13^C_G_ + *Ab*; fig. S7).

The observed ^13^C-enrichment of fatty acids, building blocks of lipids, in fungal biomass formed from ^13^C-lignin (^13^C_Lg_ + *Ab*) can be the result of de novo synthesis from the ^13^C–fatty acid impurities in the ^13^C-lignin isolate. Still, another possibility is that lipids (fatty acids) present in the unlabeled fungal inoculum were elongated, via central intermediates such as ^13^C–acetyl-CoA and ^13^C-citrate (Fig. 5), resulting, to some extent, from the ^13^C-lignin used.

Current endeavors in using lignin as source for (green) chemicals are strongly focused on microbial funneling of monomeric aromatic compounds to central intermediates to biotechnologically produce value-added platform chemicals (*36*, *37*). Within these valorization endeavors, the microbes chosen to carry out the conversions are often unicellular organisms (bacteria or yeasts) (*37*). However, we also see the need to establish parallel valorization pipelines where polymeric, insoluble, recalcitrant lignin-rich side streams are converted by multicellular organisms and/or microbial consortia, such as in the commercial *A. bisporus* production process (*38*). Deepening our understanding of the fungal conversion of lignin and the fungal metabolic pathways involved will open leads to convert lignin into metabolic intermediates and subsequently, into desired compounds and products. Furthermore, in environmental sciences, understanding of lignin bioconversion into microbial biomass, as shown by our research, is highly relevant, especially in light of terrestrial carbon cycling and sequestration.

## MATERIALS AND METHODS

### Chemicals

All chemicals were of analytical grade and purchased from Sigma-Aldrich or Merck unless specified otherwise. Water was purified using a MilliQ system (Millipore, Billerica, MA, USA).

### Isolation of high-purity lignin from nonlabeled and uniformly ^13^C-labeled wheat straw

Nonlabeled (“^12^C”, 98.9 atom % ^12^C) and uniformly ^13^C-labeled (“^13^C”, 97.7 atom % ^13^C) spring wheat plants (*Triticum aestivum* L. cv. “Baldus”), grown and provided by IsoLife bv (Wageningen, The Netherlands), had previously been planetary ball milled and sequentially water and dioxane extracted as reported (*18*). Here, lignin was respectively obtained from the reported nonlabeled and uniformly ^13^C-labeled insoluble residues by mild dioxane acidolysis and subsequent purification as described in detail in the Supplementary Materials.

### *A. bisporus* inoculum preparation

*A. bisporus* (strain A15, provided by Plant Breeding, Wageningen University, The Netherlands) was precultured on malt extract agar overlaid with autoclaved milliQ-swollen cellophane and incubated (static, 25°C). After 5 days, the mycelium was peeled off and blended in autoclaved minimal medium in an autoclaved Waring blender for 0.25 min at full speed. Antibiotics (3.5 U of streptomycin sulfate and 750 U of penicillin G sodium salt) to prevent bacterial contamination and vitamins [0.5 μM thiamine and 0.1 μM D(+) biotine] were sterile filtered and added to the blended mycelium slurry.

### Cultivation of *A. bisporus* in submerged batch fermentations

Nonlabeled and ^13^C-isotope–labeled lignin substrate (table S1) and other carbon sources (glucose and arabinose/xylose) were accurately weighed in 50-ml wide-neck Erlenmeyer flasks filled by 15.8 to 20 ml. Liquid minimal media contained 2 mM KH_2_PO_4_, 1 mM MgSO_4_, 0.5 mM CaCl_2_, 0.134 mM EDTA, 25 μM FeCl_3_, 5 μM ZnSO_4_, 5 μM MnSO_4_, 4.8 μM H_3_BO_3_, 2.4 μM KI, 52 nM Na_2_MoO_4_, 4 nM CuSO_4_, 4 nM CoCl_2_, and 20 mM (NH_4_)H_2_PO_4_ and were added to lignin and carbohydrate substrates, all reaching final concentrations of 1.5 g liter^−1^, except in the ^12^C*_Lg_, where arabinose (0.043 g liter^−1^), xylose (0.018 g liter^−1^), glucose (0.01 g liter^−1^), and lipids (0.006 g liter^−1^) (all ^13^C-isotope labeled) were added to nonlabeled lignin substrate at levels present in ^13^C-isotope lignin. Before autoclaving, the lignin-containing flasks were stirred (room temperature, 2 hours, 600 rpm) and sonicated (1 min; CPX2800H, Branson Ultrasonics, Brookfield, CT, USA) to suspend the insoluble substrate. After these medium treatment steps, lignin was partly solubilizing but the main part was insoluble (about 65% of total lignin)*.* After cooling the autoclaved flasks to room temperature, 1 ml of blended mycelium was aseptically added to all flasks and closed with autoclaved permeable lids (Polyurethane Foam Stoppers, Thermo Fisher Scientific, Bremen, Germany). One milliliter of blended mycelium slurry was lyophilized in triplicate to determine the mycelial seed dry weight. The inoculated flasks were incubated (25°C) and manually agitated on a daily basis to resuspend the lignin substrate and the mycelium.

### Harvesting *A. bisporus* biomass and fractionation of residual lignin substrate

After 15 days of incubation, the entire content of the flasks was decanted through disposable sieves (EASYstrainer, 100-μm mesh, Greiner Bio-One). The filter cake was composed of fungal biomass, although still (based on prior experimental experiences) more than 50% (w/w) of the filter cake was residual lignin substrate. Washing of the fungal biomass with water (10 ml) helped to remove loosely attached lignin, although densely incorporated and tightly attached lignin substrate was impossible to remove. Therefore, the washed fungal biomass (with incorporated lignin) was lyophilized, weighed, milled, and the lignin content and composition was determined by pyrolysis–GC-MS. The water washes were pooled with the remaining filter flow through. The flow through contained residual soluble and insoluble lignin substrate, as the pore size of the sieve mesh allowed the suspended lignin substrate to pass. Next, the combined fraction was separated in a water-soluble and insoluble fraction after centrifugation (18,000*g*, 30 min, 20°C). The pellet was washed with water three times (18,000*g*, 30 min, 20°C) and the wash liquids were combined. The residual washed insoluble lignin pellet was lyophilized (LR), as were the combined liquids containing the soluble lignin populations (LS), and carefully weighed. LR was subjected to lignin content and structural determination by pyrolysis–GC-MS and HSQC NMR and carbohydrate analysis. LS was subjected to carbohydrate analysis, and an aliquot of LS rest was further purified to separate remaining salts from (degraded) lignin fragments. Hereto, solid-phase extraction (SPE) was applied, using a reversed-phase (C18) SPE. Sep-Pak C18 cartridges (6 cm^3^, 1 g, Waters Corp., Milford, MA, USA) were activated with 10 ml of 100% (v/v) methanol (MeOH) and then washed with 20 ml of milliQ. All LS fractions were resolubilized in milliQ and quantitatively loaded on the C18 cartridges and washed with milliQ (5 ml; five times). Next, C18-bound compounds were eluted with 100% MeOH (5 ml; two times), pooled, then evaporated under N_2_, and dried. The dried fractions were weighed and used to analyze the lignin content by pyrolysis–GC-MS and subjected to HSQC NMR.

### Proteomics sample preparation and analysis

#### Proteomics—sample preparation, S-trap

The ^12^C_Lg_ + *Ab* was solely cultivated to investigate secreted enzymes. The supernatants were filtered through a 0.45- and 0.2-μm Whatman filer and subsequently concentrated (IVSS Vivaspin 20, 3000 kDa). The concentrate extracellular enzymes were collected in the filtrate and subjected to proteomics.

A volume of filtrate containing ~100 μg of protein was aliquoted and SDS was added to final 5% (w/v) solution and incubated for 30 min with 10 mM tri-chloro-ethyl-phosphine and 20 mM iodo-acetamide in 50 mM tri-ethyl ammonium bicarbonate (pH 7.5) to reduce and denature the proteins. Phosphoric acid was added to 1.2% final. Subsequently, the proteins were precipitated with 80% methanol on a micro–S-trap filter device (Protifi, Fairport NY, USA, www.protifi.com). Samples were further processed according to manufacturer’s instruction. The precipitated proteins were digested with 1 μg of MS grade trypsin during overnight incubation at 37°C temperature. The digest peptides were eluted, dried, and redissolved in 0.1% formic acid (FA) in water.

#### Proteomics—chromatography and MS

The extracted digested peptides were injected and separated with a Waters M-class UPLC system (Waters Corp., Milford, MA, USA) online connected to a Qexactive^PLUS^ mass spectrometer (Thermo Fisher Scientific, Palo Alto, USA). Peptides were first collected on a trap column (20 × 15 mm; PepSepC18 Trap, PepSep, Denmark) and subsequently separated on an analytical C18 column (100 mm × 75 μm, PepSep, Denmark) with a 30-min gradient of 4 to 16% and 15-min gradient to 25% acetonitrile in 0.1% FA followed by a column cleanup step (isocratic 80% acetonitrile in 0.1% FA) and an equilibration step (isocratic 2% acetonitrile in 0.1% FA), all at a flow rate of 200 nl min^−1^. The eluted peptides were electrosprayed into the mass spectrometer using a Flex-Ion nanoESI Source at +2.4 kV. MS and MSMS spectra were collected in top-10 DDA mode selecting charge 2, 3, or 4 ions within mass/charge ratio (*m*/*z*) range of 400 to 1500 for MS spectra and auto-range for MSMS spectra.

#### Proteomics data processing with MaxQuant

MSMS raw data were processed using MaxQuant software version 1.6.17.0. Settings were mostly default, with variable modifications oxidation (M) and acetyl (protein N-term) and fixed modification carbamidomethyl (C). Data were matched to the *A. bisporus* H97.v2–filtered models, proteome sequences from Joint Genome Institute identifier containing 10,438 entries (*11*).

#### Proteomics analysis with Perseus

Hierarchical clustering to generate heatmaps was done with Perseus. Selection of proteins was manually curated and was based on their substrate specificities (table S2, proteomics table). A log2 transformation was carried out on IBAQ and hierarchical clustering with Euclidean distance was performed.

### Quantitative lignin analysis by pyrolysis–GC-MS

To determine the extent of delignification, the lignin contents of residual lignin substrate (LS and LR) and substrate incorporated in fungal biomass were determined by pyrolysis–GC-MS as described by van Erven *et al*. (*18*, *39*). Lignin preparations respectively isolated from ^13^C-labeled (97.7 atom % ^13^C) and nonlabeled (98.9 atom % ^12^C) wheat straw (IsoLife BV, Wageningen, The Netherlands) were used as internal standards. When ^13^C-isotope labeled lignin was used as carbon source for *A. bisporus* cultivation (^13^C_Lg_ + *Ab* and ^13^C_Lg_), the lignin internal standard was nonlabeled (^12^C-IS; internal standard), and for the nonlabeled lignin samples spiked with carbohydrates (^12^C*_Lg_ + *Ab*), the uniformly ^13^C-labeled internal standard was used.

### Structural characterization of lignin by ^1^H-^13^C HSQC NMR

HSQC NMR spectra were recorded at 25°C on a Bruker AVANCE III 600 MHz NMR spectrometer (Bruker BioSpin, Rheinstetten, Germany) equipped with a 5-mm cryo-probe located at MAGNEFY (MAGNEtic resonance research FacilitY, Wageningen, The Netherlands) and based on previously reported procedures (*40*) and is further described in Supplementary Method S2.

### Sugar content and composition of lignin isolate before and after fungal treatment

The neutral sugar content of the initial lignin substrate, and water insoluble (LR), was determined after methanolysis followed by trifluoroacetic acid (TFA) hydrolysis. Shortly, samples were methanolyzed in dried methanol for 16 hours at 80°C, which after the released methyl glycosides were hydrolyzed using 2 M TFA according to de Ruiter *et al.* (*41*). Released free monosaccharides from LR and initial lignin substrate and free sugars from LS were analyzed by high-performance anion exchange chromatography with pulsed amperometric detection with postcolumn alkali addition. Detailed information regarding the elution profile can be found in Supplementary Method S3. Before injection, samples were dissolved in MilliQ to reach individual monosaccharide concentrations of 1 to 50 μg/ml and centrifuged (20,000*g*, 10 min, 20°C). Response factors of a monosaccharide standard subjected to the same method were used for quantification. The monosaccharide standard was composed of eight sugars (arabinose, xylose, glucose, fructose, galactose, mannose, fucose, and rhamnose).

### SEM of lignin and *A. bisporus* cultivated on lignin and glucose

SEM of lignin, *A. bisporus* with lignin, and the glucose-grown *A. bisporus* were analyzed with a Magellan 400 device (FEI, Eindhoven, The Netherlands) at the Wageningen Electron Microscopy Center. The lignin and *A. bisporus* mycelium samples were dried on 13-mm filters with 10-μm pores [Merck Isopore membrane filter (Merck, Burlington, MA, USA)], attached to sample holders containing carbon adhesive tabs (EMS, Washington, USA) and sputter coated with 12-nm tungsten (EM SCD 500, Leica, Wetzlar, Germany). SEM images were recorded at an acceleration voltage of 2 kV and 13 pA and magnification of 250, 1000, 5000, and 10,000 times using the Everhart-Thornley detector.

### ^13^C-isotope enrichment analysis of amino acids

For the analysis of proteinogenic amino acids, fungal biomass (1 mg) was hydrolyzed by incubation in 100 μl of 6 M HCl for 24 hours at 100°C and clarified from cell debris by filtration (0.2 μm, Ultrafree-MC, Merck Millipore, Darmstadt, Germany). Subsequently, the hydrolysate was dried under a nitrogen flow. The obtained amino acids were dissolved in 50 μl of *N*,*N*-dimethylformamide containing 1% (v/v) pyridine and derivatized at 80°C for 30 min with 50 μl of *N*-methyl-*t*-butyldimethylsilyl-trifluoroacetamide (Macherey-Nagel, Düren, Germany) (*42*). Mass isotopomer distributions (MIDs) of the *t*-butyldimethylsilyl amino acids were analyzed by GC-MS (Agilent 7890A, Quadrupole Mass Selective Detector 5975C, Agilent Technologies). Before MS analysis, analytes (0.2-μl injection volume) were separated on an HP-5MS column (30 m, 250 μm × 0.25 μm, Agilent Technologies) using helium as the carrier gas (1.7 ml min^−1^) and the following temperature program: 120°C (0 to 2 min), 8°C min^−1^ increase (2 to 12 min), 10°C min^−1^ increase (12 to 24.5 min), and 325°C (24.5 to 27 min). Further settings controlled the inlet (250°C), transfer liner (280°C), ion source (230°C), and quadrupole temperature (150°C). Samples of natural glucose- and lignin-grown biomass were first measured in scan mode to exclude potential isobaric interference of the complex sample matrix with ion clusters of interest. Subsequently, selective ion monitoring (SIM) was used to obtain MIDs from fragments that either contain the entire amino acid carbon skeleton or fragments in which C_1_ is released during ionization. Together, 15 amino acid fragments yielded ion clusters with clean MIDs: alanine (C_1–3_, *m*/*z* 260), glycine (C_1–2_, *m*/*z* 246), valine (C_1–5_, *m*/*z* 288), leucine (C_2–6_, *m*/*z* 200), isoleucine (C_2–6_, *m*/*z* 200), proline (C_2–5_, *m*/*z* 258), methionine (C_1–5_, *m*/*z* 320), serine (C_1–3_, *m*/*z* 390), threonine (C_1–4_, *m*/*z* 404), phenylalanine (C_1–9_, *m*/*z* 336), aspartate (C_1–4_, *m*/*z* 418), glutamate (C_1–5_, *m*/*z* 432), lysine (C_1–6_, *m*/*z* 431), arginine (C_1–6_, *m*/*z* 442), and tyrosine (C_1–9_, *m*/*z* 466). Glutamate and aspartate also reflected the pools of glutamine and asparagine, which underwent deamination during acidic hydrolysis. The remaining proteinogenic amino acids (cysteine, histidine, and tryptophan) were not available due to their degradation during hydrolysis or a low signal-to-noise ratio. MIDs were corrected for natural occurring isotopes and were used to calculate a summed fractional ^13^C labeling (SFL) for each amino acid (*43*, *44*). Enrichments were obtained from two biological duplicates.

### ^13^C-isotope enrichment analysis of sugars

For the analysis of fungal cellular sugars, fungal biomass (2.5 mg) was hydrolyzed in 250 μl of 2 M HCl for 2 hours at 100°C. Afterward, cell debris was removed by filtration (0.2 μm, Ultrafree-MC, Merck Milli-pore). Subsequently, the hydrolysate was dried under nitrogen. Analytes contained in the dried residue were incubated in 50 μl of methoxylamine (2% in pyridine) at 80°C for 1 hour. The obtained *O*-methyloxime forms of the analytes were silylated at 80°C for 30 min into trimethylsilyl derivatives in a second step using 50 μl of *N*,*O*-bis-tri-methylsilyl-trifluoroacetamide (Macherey-Nagel) (*42*). The derivatized analytes were analyzed by GC-MS (Agilent 7890A, Quadrupole Mass Selective Detector 5975C, Agilent Technologies). In the same way, purified biopolymers (glycogen from oysters, peptidoglycan from *Bacillus subtilis*, and lipopolysaccharides from *Pseudomonas aeruginosa*, all from Sigma-Aldrich, Taufkirchen, Germany) and pure glucose, glucosamine, *N*-acetyl-glucosamine, and mannose were treated as controls for GC-MS analysis. Before MS analysis, the analytes (0.2-μl injection volume) were separated on an HP-5MS column (30 m, 250 μm × 0.25 μm, Agilent Technologies) using helium as the carrier gas (1.7 ml min^−1^) and the following temperature program: 150°C (0 to 3 min), 8°C min^−1^ increase (2 to 12 min), 25°C min^−1^ increase (12 to 15.8 min), and 325°C (15.8 to 19 min). Further settings controlled the inlet (250°C), transfer liner (280°C), ion source (230°C), and quadrupole temperature (150°C). Again, control samples were first measured in scan mode to exclude isobaric interference of the lignin matrix with signals from ion clusters of interest. Subsequently, SIM was used for quantitative analysis. The following sugar(amine) fragments were analyzed, corrected for natural occurring isotopes and used to determine their SFL: ribose (C_3–5_, *m*/*z* 307 and C_1–5_, *m*/*z* 467), glucose (C_3–6_, *m*/*z* 319 and C_1–6_, *m*/*z* 554), and glucosamine (C_3–6_, *m*/*z* 319 and C_1–6_, *m*/*z* 553). Enrichments that were obtained from two biological duplicates lastly considered for flux estimation are listed in table S10.

### ^13^C-isotope enrichment analysis of fatty acids

For the analysis of ^13^C-enriched fatty acids, fungal biomass (10 mg) was used, and lipids were extracted according to modified protocols Dodds *et al.* (*45*) and ^13^C-isotope fractional enrichment was carried out according to Pi *et al.* (*46*). Freshly prepared chloroform/methanol mix [4 mL, 2:2.5 (v/v)] containing 1,2-diundecanoyl-*sn*-glycero-3-phosphocholine (8.5 μg ml^−1^; PC11:0) was added to the fungal biomass, serving as internal standard. The suspension was bead beaten (1 min, 10 m s^−1^; FastPrep-24 5G, MP Biomedicals Asia Pacific) and sonicated (10 min). Then, 2.5 ml of tris buffer (50 mM tris and 1 M NaCl, pH 7.5) was added, followed by vortexing (5 s), sonicating (10 min), and centrifuging (5 min, 1174*g*, 15°C). The lower biomass-free chloroform phase was carefully transferred to a tube. The residues were re-extracted three times in chloroform (1 mL). All chloroform-containing supernatants were pooled and dried under N_2_. The dry pellets were resolubilized in toluene (0.3 ml), and methanol (2.7 ml) with 5% H_2_SO_4_ was added for methylation and incubated (1 hour, 100°C). After cooling to room temperature, milliQ (3 ml) and hexane (3 ml) spiked with methyl nonadecanoate (5 μg ml^−1^; 19:0; a second internal standard) were added. The samples were rotated head-over-tail (15 min) and centrifuged (5 min, 1174*g*, 15°C). A volume of 1 μl of the derivatized sample was injected in a gas chromatograph (Trace1300, Thermo Fisher Scientific, Waltham, MA, USA) equipped with a Restek FAMEWAX column (30 m × 0.25 mm inside diameter and film thickness 0.25 μm) coupled to a mass spectrometer (Exactive Orbitrap, Thermo Fisher Scientific, Waltham, MA, USA), allowing sensitive and accurate (five-digit) determination of masses. The split injection with 20 ml min^−1^at 150 kPa was used with the injection port held at 250°C. The initial oven temperature was set to 150°C for 2 min, ramped to 250°C at a rate of 4.0°C min^−1^, and held at 250°C for 3 min. The linear velocity of the carrier gas helium was constant at 10 ml min^−1^. The MS was operated at positive mode, full scan (*m*/*z* 50 to 400). For fatty acid quantification, flame ionization detection was used. Peak integration was carried out with a processing method by TraceFinder 5.1, and wrongly integrated peaks were manually corrected. Fatty acid methyl standards (CRM18920, Supelco, Bellefonte, PA, USA) were used for fatty acid annotation and quantification. Individual calibration curves for FAs were in the range between 0.09 to 0.6 and 9.12 to 62.4 μg ml^−1^. The averaged slopes of the calibration curves were used as RF. The methyl nonadecanoate was used as a system check internal standard. Calibration and calculation of fatty acid content was carried out according to Dodds *et al*. (*45*). Subsequently, selective ion extraction was used to determine the MIDs of fatty acids, which were made up more than 2.5% (w/w) (table S8 and fig. S5), and example mass spectra are shown in fig. S6. The mass fragments used for MID are listed in table S9 and fig. S5. The MIDs were calculated according to Pi *et al.* (*46*).

### ^13^C-isotope enrichment analysis of ergosterol

To determine the ^13^C-fractional enrichment of ergosterol, fungal biomass was analyzed by pyrolysis–GC-MS with settings as described by van Erven *et al.* (*39*), the same settings as for lignin quantification were used but conversely, without response factor correction. MIDs were calculated as described for fatty acids and the selected ions are listed in the table S8.

## Supplementary Materials

### This PDF file includes:

Supplementary Methods S1 to S3 Figs. S1 to S8 Tables S1 to S10 Legend for data S1 References
