# Supplementary material for: From 13C-lignin to 13C-mycelium: Agaricus bisporus uses polymeric lignin as a carbon source
Source: Sci Adv. 2024 Apr 19;10(16):eadl3419. doi: 10.1126/sciadv.adl3419 (PMC11029805; doi:10.1126/sciadv.adl3419)
Supplement: Supplementary file 1 — Supplementary Methods S1 to S3 Figs. S1 to S8 Tables S1 to S10 Legend for data S1 References [file sciadv.adl3419_sm.pdf]

Science Advances
AAAS

Supplementary Materials for

From ^{13}C-lignin to ^{13}C-mycelium: *Agaricus bisporus* uses polymeric lignin as a carbon source

Katharina Duran *et al.*

Corresponding author: Mirjam A. Kabel, mirjam.kabel@wur.nl

Sci. Adv. **10**, eadl3419 (2024)
DOI: 10.1126/sciadv.adl3419

The PDF file includes:

Supplementary Methods S1 to S3
Figs. S1 to S8
Tables S1 to S10
Legend for data S1
References

Other Supplementary Material for this manuscript includes the following:

Data S1

Supplementary Text

Supplementary Method 1: Lignin isolation for labelling studies

Non-labelled ('^{12}C', 98.9 atom % ^{12}C) and uniformly ^{13}C-labelled ('^{13}C', 97.7 atom % ^{13}C) straw from spring wheat plants (*Triticum aestivum* L. cv. 'Baldus') grown and provided by IsoLife (Wageningen, The Netherlands) had previously been planetary ball-milled and sequentially water and dioxane extracted as reported (*18*). Four portions of approximately 500 mg of the respective nonlabelled and uniformly ^{13}C-labelled reported dioxane insoluble residues were accurately weighed in glass reaction tubes, mixed with 10 mL 90% v/v aqueous dioxane containing 0.25 M HCl, purged with N_2 and vortexed thoroughly before addition to a Stuart SBH200D/3 heating block (Cole Palmer, Vernon Hills, IL, USA) at 100 °C. Tubes were vortexed every 5 min for a total reaction time of 60 min. After the treatment, the tubes were cooled on ice and centrifuged (2500 x *g*, 2 min, 20 °C) to separate the soluble and insoluble fractions. The four respective supernatants and residues were combined. Combined residues were washed twice with 5 mL 90% aqueous dioxane and soluble fractions obtained through centrifugation (2500 x *g*, 2 min, 20 °C) were added to the initial supernatants. Combined supernatants were transferred to 400 mL cold water, vigorously shaken, and left to precipitate at 4 °C for 2 h. Formed precipitates were obtained by centrifugation (18.000 x *g*, 5 min, 20 °C), washed twice with 30 mL water and freeze-dried. Dried precipitates were redissolved in 5 mL ethanol/chloroform 50:50 v/v, vortexed for 1 min, and precipitated in 40 mL cold petroleum ether and left to precipitate at 4 °C for 1 h. Precipitates were obtained through centrifugation (2500 x *g*, 2 min, 20 °C), washed once with 40 mL cold petroleum ether and dried under nitrogen flow at room temperature to yield the final purified non-labelled ($^{12}C_{LG}$) and uniformly ^{13}C labelled lignin isolates ($^{13}C_{LG}$), respectively.

Supplementary Method 2: Structural characterization of lignin by ^{1}H-^{13}C HSQC NMR

HSQC NMR spectra were recorded at 25 °C on a Bruker AVANCE III 600 MHz NMR spectrometer (Bruker BioSpin, Rheinstetten, Germany) equipped with a 5 mm cryo-probe located at MAGNEFY (MAGNEtic resonance research FacilitY, Wageningen, The Netherlands) and based on previously reported procedures (*18*). Purified soluble (LS), and water-insoluble (LR) residual lignin from $^{13}C_{LG}$+*Ab* and $^{13}C_{LG}$ (0.25 mg) were dissolved in 0.6 mL DMSO-d_6 and transferred to NMR tubes, though the water-insoluble fungal-treated samples were not completely soluble. The spectra were recorded using the "hsqcetgpsisp2.2" pulse program. In the ^{1}H dimension a spectral width of 12 ppm, an offset of 4.7 ppm, and 2048 increments were used, and in the ^{13}C dimension a spectral width of 200 ppm, an offset of 100 ppm, and 400 increments were used. Sixteen scans were recorded by using a recycle delay (D1) of 0.86 s, and a $^{1}J_{CH}$ of 145 Hz. Spectra were processed using Gaussian apodization (GM; GB = 0.001, LB = −0.2 Hz) in ^{1}H and a squared sine function (QSIN; SSB = 2) with 1024 increments in both ^{1}H and the ^{13}C dimensions.

The solvent peak (DMSO-d_6) was used as an internal chemical shift reference (δ_C 39.5 ppm; δ_H 2.49 ppm) for processing Bruker TopSpin v4.0.5 was used. Phase correction was done manually and baseline correction was done automatically. Correlation peaks were assigned by comparison with literature (*40, 49, 50, 51, 52*). Semi-quantitative analysis of the HSQC volume integrals was performed according to Del Río (*18*). $S_{2,6}$, G_2 and $H_{2,6}$ signals were used for S, G and H units, respectively, where S and H integrals were halved. The oxidized analogues were semi-quantified in a similar manner. Tricin, *p*CA and FA were similarly semi-quantified from their respective $T_{2',6'}$, $p\mathrm{CA}_{2,6}$ and FA_2 signals. The β-*O*-4′ substructures were semi-quantified by their C_β-H_β correlations, whereas β-5 and β-β substructures were semi-quantified on their C_α-H_α correlations Volume integrals for resinol substructures were halved. Dihydroxypropiovanillone and

dihydroxypropiosyringone (DHPV/S), hydroxypropiovanillone and dihydroxypropiosyringone (HPV/S), were semi-quantified from their C_β-H_β correlations. Benzaldehydes and cinnamaldehydes were quantified on their C_α-H_α correlations and cinnamyl alcohol was quantified on C_γ-H_γ correlations. Volume integrations were performed at similar contour levels, and between spectra the contour levels were normalized to an equal size of $–OCH_3$ integral. Substructures were expressed per 100 aromatic rings ($H + G + G_{ox} + S + S_{ox}$).

Supplementary Method 3: Sugar content and composition of the lignin isolates before and after fungal treatment

For the quantification of (released) monosaccharides an ICS-5000 HPLC system (Dionex, Sunnyvale, CA, USA) equipped with a CarboPac PA1 guard column (2 mm ID ×50 mm) and a CarboPac PA-1 column (2 mm ID ×250 mm; both from Dionex) was used for analysis. The detection of the eluted monosaccharides was performed by an ED40 EC-detector running in the PAD mode (Dionex). 10 µL of the 50 times diluted hydrolysates (LR and initial substrate), or non-hydrolyzed soluble fractions (LS) were injected on the system. Mobile phases used to elute the compounds were kept under nitrogen, and the column temperature was set at 20 °C. A flow rate of 0.4 $mLmin^{-1}$ was used with the following elution profile of 0.1 M sodium hydroxide (NaOH: A) and 1.0 M NaOAc in 0.1 M NaOH (B): 0−35 min, 100 % milli-Q water; 35.1 min, 100 % A; 35.2−50 min, 0–40 % B; 50.1−55 min, 100 % B; 55.1–63.0 min, 100 % A; 63.1–78.0 min, 100 % milli-Q water. A post-column alkali addition (0.5 M NaOH; 0.1 mL/min) was used from 0.0 to 34.9 min and from 68.1 to 78.0 min. All samples were analyzed in duplicate. The collected data was analyzed using Chromeleon 7.2 software (Dionex Corporation).

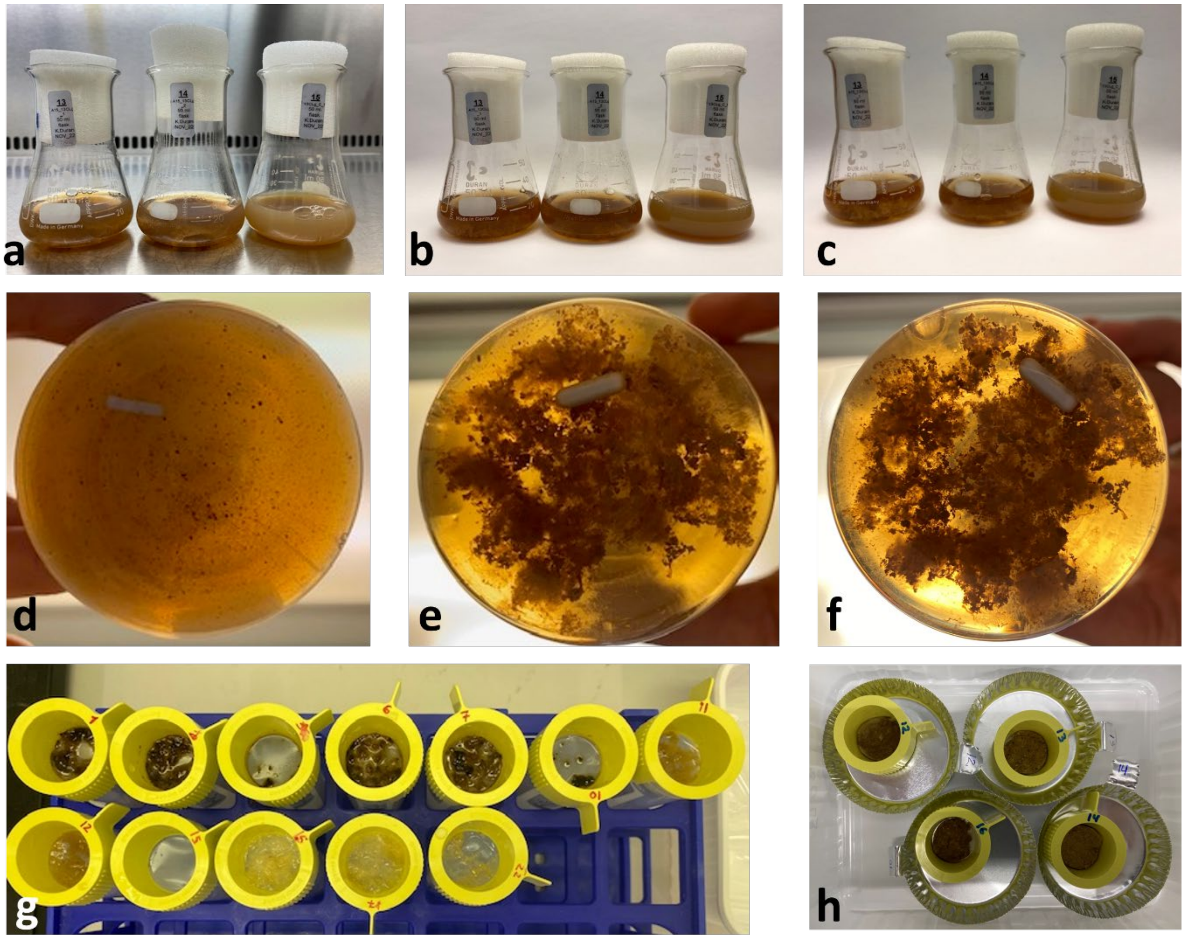

Fig. S1

^{13}C-wheat straw lignin isolate control and *Agaricus bisporus* cultivated on ^{13}C-wheat straw lignin isolate ($^{13}C_{LG}$ and $^{13}C_{Lg}$+*Ab*). Fermentation flasks after 5 days (a), 10 days (b), and 15 days (c). The flasks from below show the uninoculated control $^{13}C_{LG}$ (d), and the biological duplicates of $^{13}C_{Lg}$+*Ab* (e, f). The harvested fungal biomass was collected in sieves (g), and lyophilized (h).

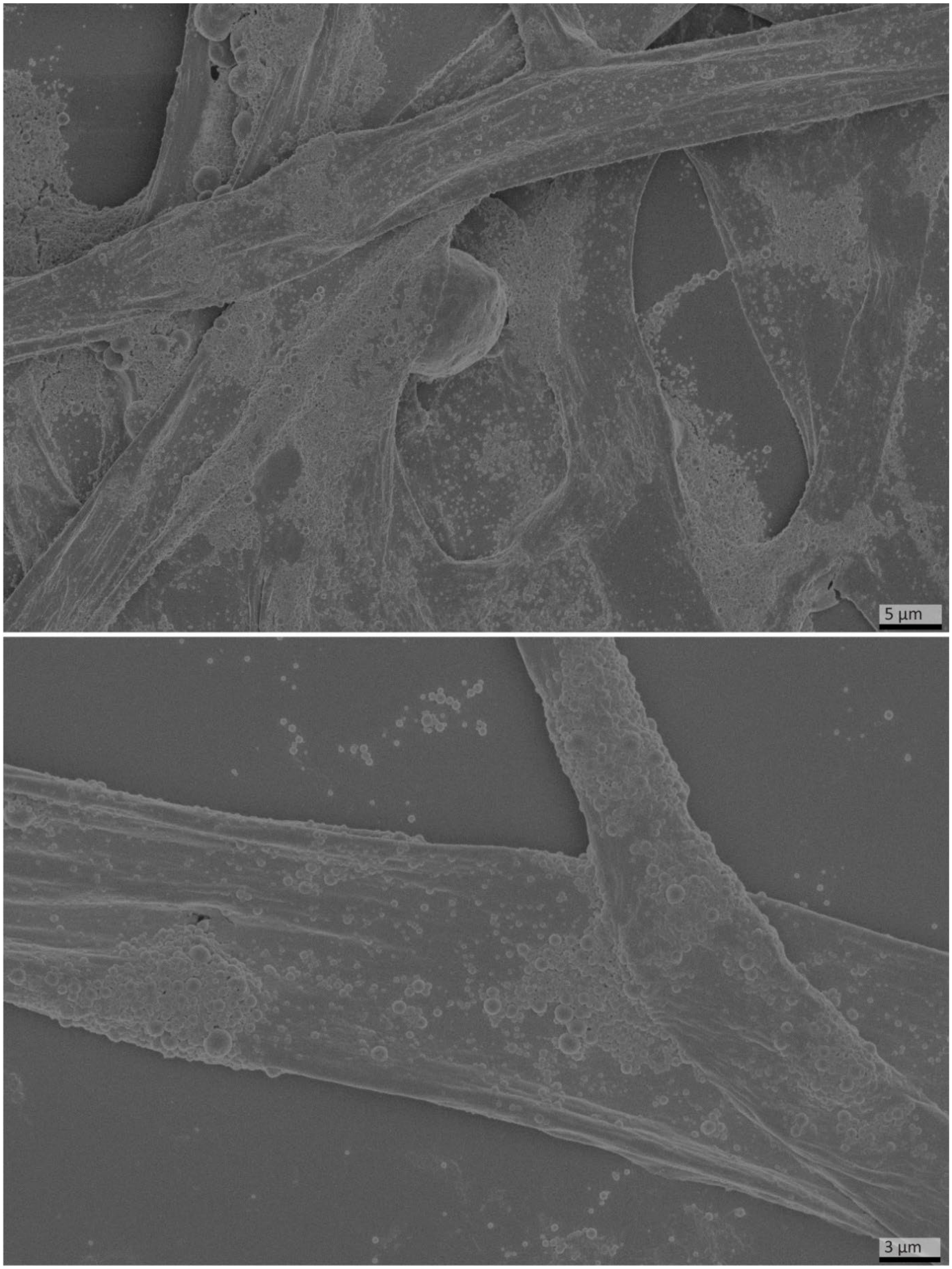

Fig. S2

Scanning electron microscope (SEM) pictures of *A. bisporus* grown on lignin. Lignin is visible as ‘particles’ on top of the fungal hyphae.

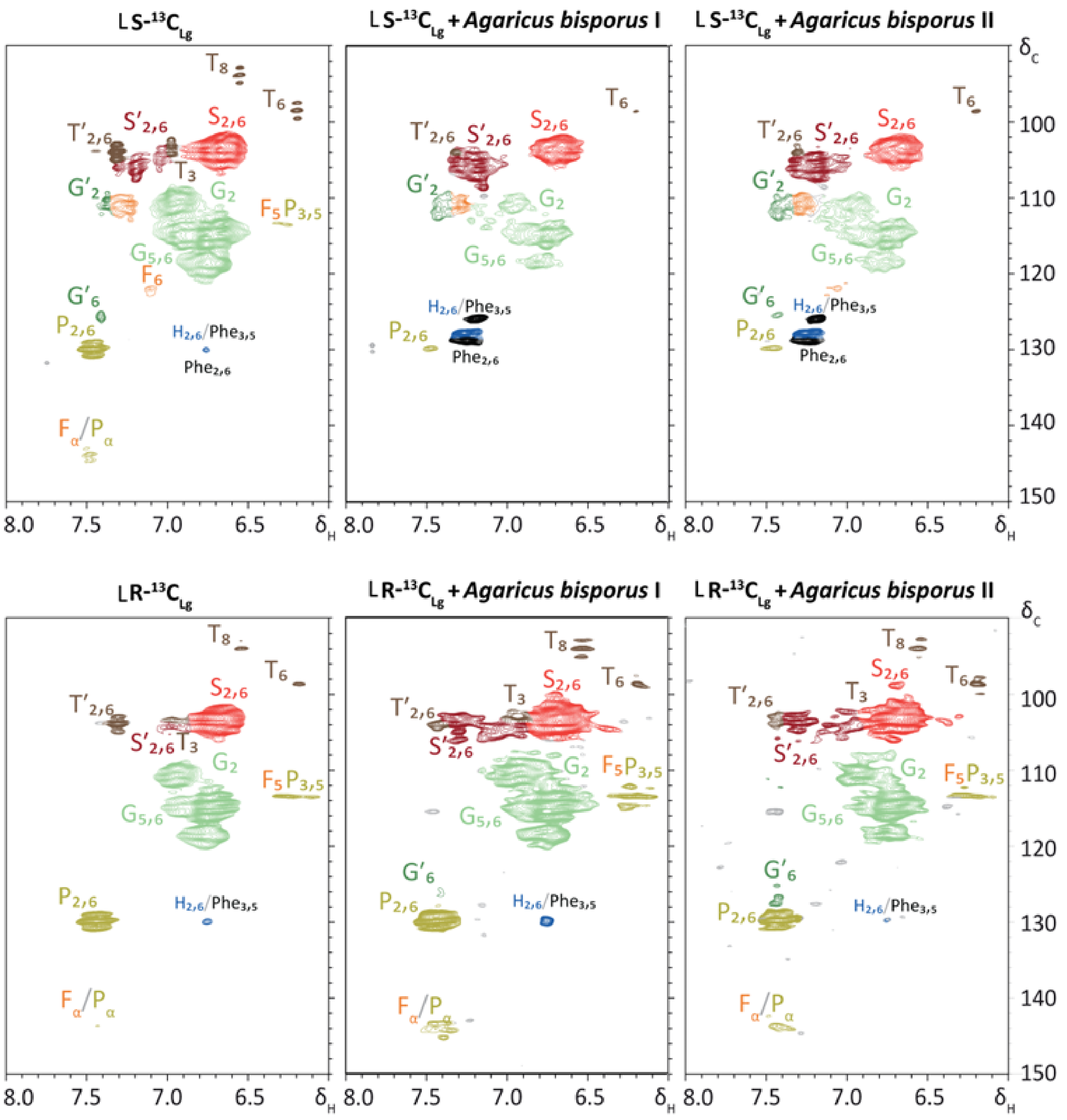

Fig. S3

Aromatic regions of ^{1}H-^{13}C HSQC NMR spectra of water soluble (LS) and insoluble (LR) residual lignin after fungal treatment of biological duplicates ($^{13}C_{Lg}$+*Agaricus bisporus* I, $^{13}C_{Lg}$+ *Agaricus bisporus* II,) compared with the control ($^{13}C_{LG}$). Colour codes of spectra correspond to structures Figure 3 in main text and grey represents unassigned spectra. See supporting information for tables containing semiquantitative analysis of volume integrals (Supplementary Table 7). For explanation of sample codes see Figure 1 in main text.

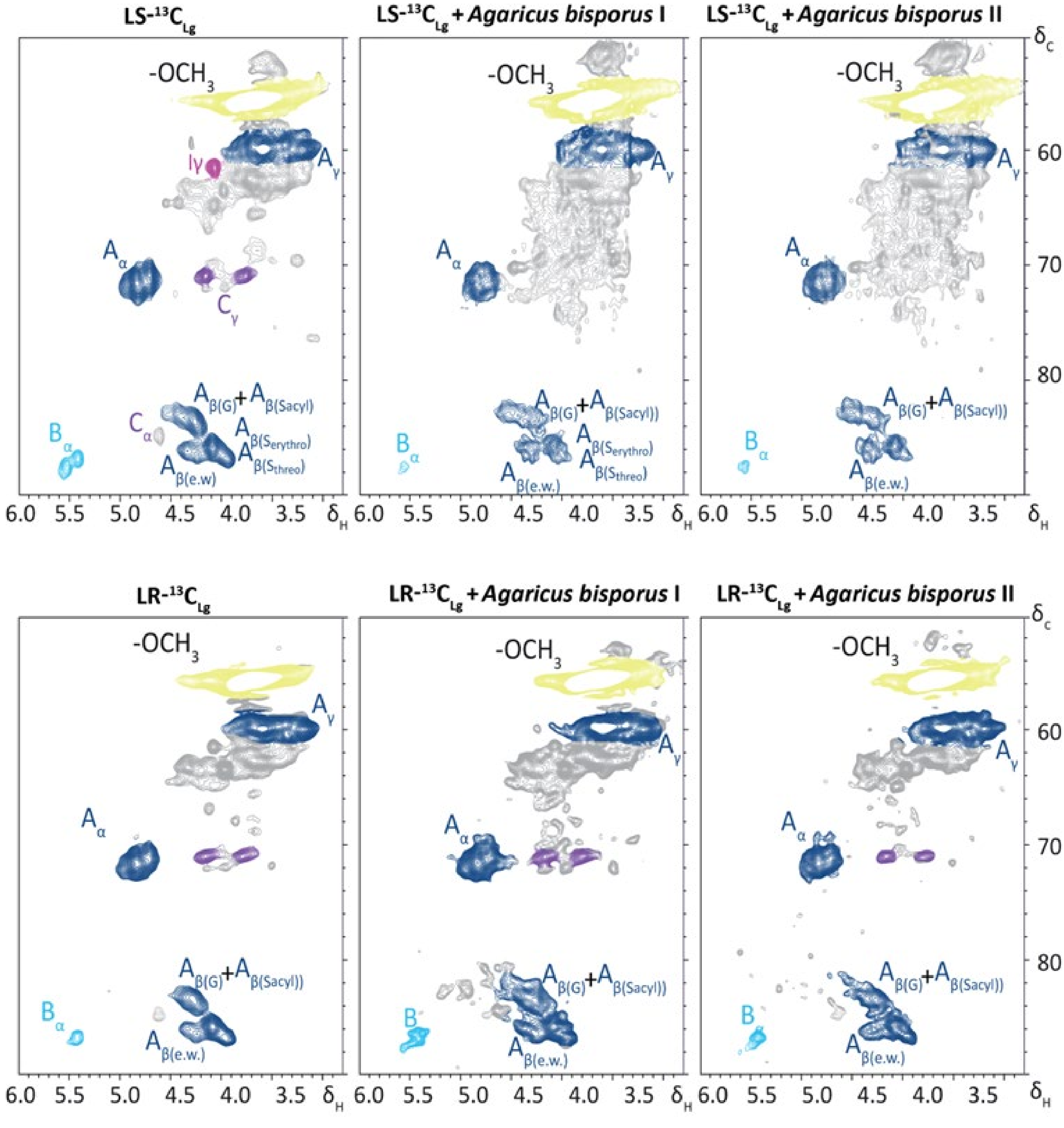

Fig. S4

Aliphatic regions of ^{1}H-^{13}C HSQC NMR spectra of water soluble (LS) and insoluble (LR) residual lignin after fungal treatment of biological duplicates ($^{13}C_{LG}$+*Agaricus bisporus* I, $^{13}C_{Lg}$ + *Agaricus bisporus* II,) compared with the control ($^{13}C_{LG}$). Colour codes of spectra correspond to structures Figure 3 in main text and grey represents unassigned spectra. See supporting information for tables containing semiquantitative analysis of volume integrals (Supplementary Table 7). For explanation of sample codes see Figure 1 in main text.

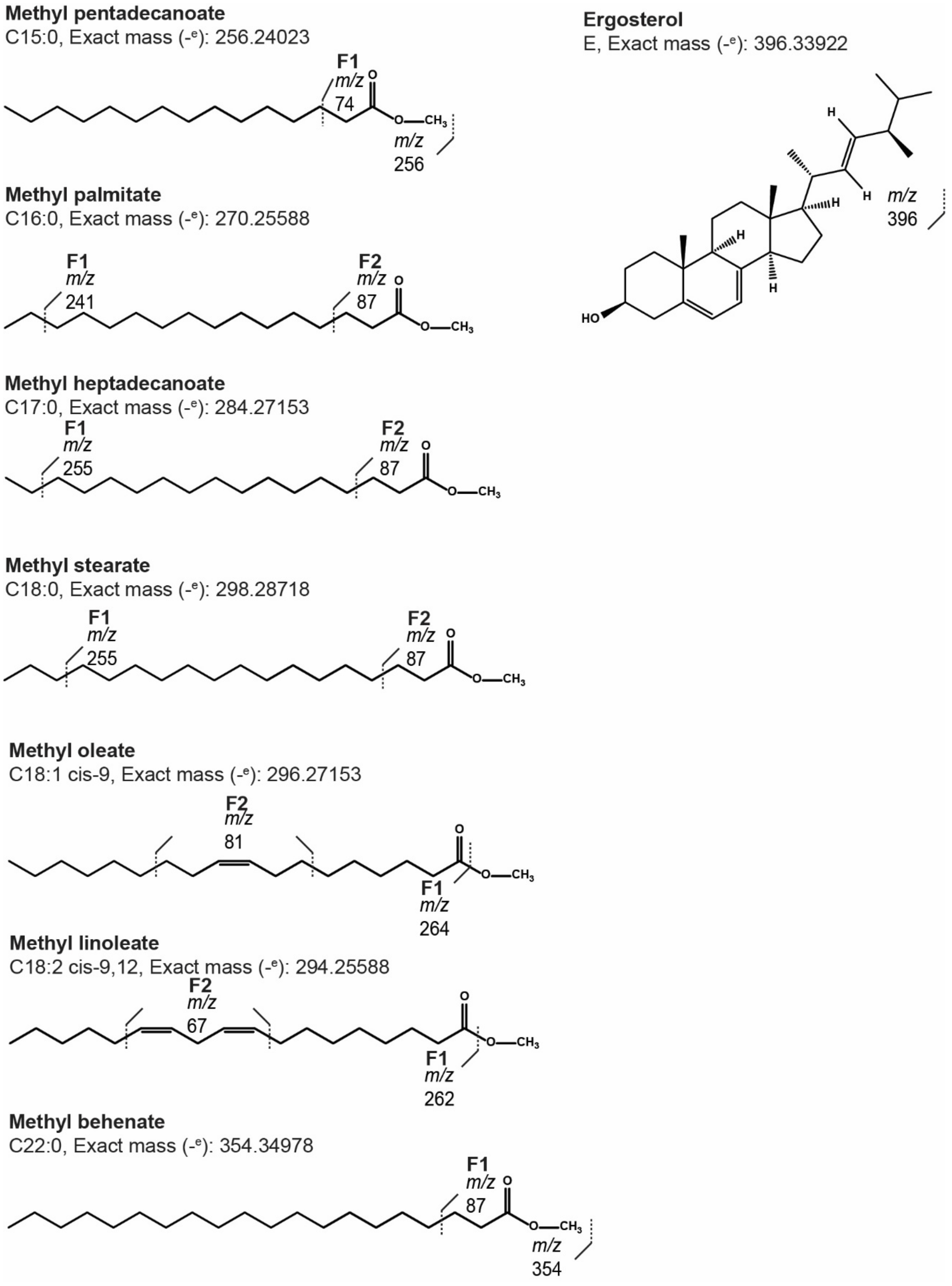

Fig. S5

Chemical structures of fatty acid methyl esters and ergosterol with exact masses (Orbitrap MS) with indicated fragmentations and *m/z* that were selected for fractional labelling.

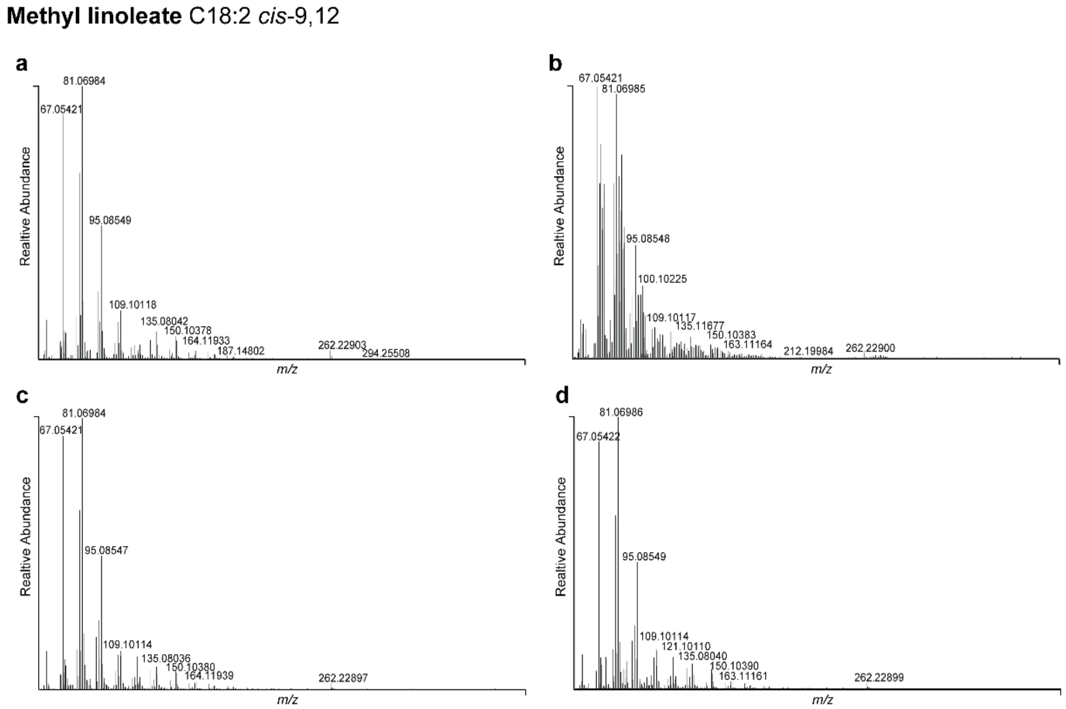

Fig. S6.

Mass spectra of methyl linoleate (C18:2 cis 9,12) at retention time of 13.7 min of standard (a), ^{13}C glucose grown *Agaricus bisporus* (b), ^{13}C lignin grown *A. bisporus* (c) and ^{12}C lignin spiked with ^{13}C carbohydrates and lipids grown *A. bisporus* (d).

Table S1

Composition (based on dry matter) of the purified ^{13}C-lignin isolate from wheat straw ($^{13}C_{Lg}$; see Figure 1), including the fatty acid composition (mol%).

Compounds	Content (w/w%)
**Carbohydrates**[a]	
Rhamnose	0.0
Fucose	0.0
Arabinose	3.5
Xylose	1.4
Mannose	0.0
Galactose	0.0
Glucose	0.8
Uronic acid	0.2
Glucosamine	0.0
**Sum total carbohydrates**	6.1
**Lignin**[b]	93.0
**Protein**[c]	Traces (<0.5)
**Fatty acids**	0.5
**Sum of compounds analyzed**	100.1
**Fatty acid composition**	**mol %**
C12:0	0 ± 0
C13:0	0 ± 0
C14:0	0 ± 0
C15:0	0 ± 0
C16:0	42.2 ± 1.6
C16:1, cis	0 ± 0
C17:0	0 ± 0
C18:0	14.6 ± 1.5
C18:1, *cis/trans* 9 mix	19.9 ± 11.1
C18:2, *cis* 9,12	0 ± 0
C20:0	23.3 ± 8
C20:1, *cis* 11	0 ± 0
C22:0	0 ± 0

[a] Shown as anhydrosugars, based on dry matter (w/w%).
[b] Lignin content analyzed by pyrolysis-GC-MS, employing a nonlabelled lignin isolate with known lignin content as internal standard
[c] Protein content estimated based on the tryptophan pyrolysis marker indole, by comparison to a wheat straw sample with known protein content of around 3.5% w/w based on nitrogen analysis (N x 6.25)., For the lignin isolate an 85% lower intensity of indole was determined.

Table S2

Fatty acid content and composition of the mixture of ^{13}C lipids used in the ‘spiked’ treatments ($^{12}C*_{Lg}$+*Ab*).

**Fatty acid**	% w/w
C12:0	0.0
C13:0	0.0
C14:0	4.5
C15:0	0.4
C16:0	43.8
C16:1, c	10.3
C17:0	1.5
C18:0	1.4
C18:1, t/c 9 mix	15.4
C18:2, c 9,12	16.2
C20:0	0.0
C20:1, c 11	0.2
C22:0	0.0

Table S3

Agaricus bisporus carbon use efficiencies (CUEs) of various lignin isolates obtained from different sources, lignin purity, carbon, and fermentation parameters used. The fermentations were set-up as described in the main manuscript. NA = not analysed.

**Lignin isolates (LI)**	**Lignin purity (w/w %)**	**[Glucose][b] (g/L)**	**[LI][b] (g/L)**	**Fermentation (days)**	**Biological replicates**	**CUE[c] (g/g)**
LI - wheat straw[d]	90.0[a]	0	0.75	14	2	0.13 ± 0.04
		0	1.5	14	4	0.12 ± 0.08
		0	1.5	35	2	0.14 ± 0.02
Soda P1000[e]	85.1	0	0.5	14	1	0.15
		0	1.5	14	1	0.01
		0	2.5	14	1	0
LI - corn stover[f]	NA	0	1.5	14	2	0.14 ± 0.08
		0	1.5	35	2	0.17 ± 0.01
		0.15	1.5	14	2	0.13 ± 0.02
		0.15	1.5	35	2	0.20 ± 0.02
^{13}C-LI - corn stover[g]	NA	0	1.5	35	1	0.2

[a] Lignin purity based on pyrolysis-GC-MS.

[b] Concentration of glucose and the lignin isolate (LI) prepared for fermentation.

[c] Carbon use efficiencies (CUEs) based on amount of mycelium biomass formed (corrected for incorporated lignin and mycelial seed) and absolute amount of lignin isolate added at the start of the fermentation Note that fungal biomass was washed with chloroform/ethanol 50:50 (v/v) to remove loosely bound lignin, which was different than the water washing step described in the Methods section.

[d] Lignin isolated from wheat straw as described by van Erven et al. (2020) (*53*) available in our lab from previous research.

[e] Soda P1000 lignin as described by Constant et al. (2016) (*54*), kindly provided by Richard Gosselink (WFBR).

[f] Soda lignin isolated from nonlabelled corn stover (IsoLife, Wageningen, The Netherlands), purified by extensive sequential solvent extraction and precipitation.

[g] Soda lignin isolated from uniformly labelled ^{13}C-corn stover (IsoLife, Wageningen, The Netherlands), purified by extensive sequential solvent extraction and precipitation.

Table S4

Lignin content, analyzed by py-GC-MS, and relative abundance of pyrolysis products of the water insoluble lignin fractions (LR) corrected for RRF *(18).* See figure 1 for fractionation scheme and sample codes. Sum on the bases of previous structural classification (*18, 40, 55*). Average and standard deviation were derived from technical duplicates. I and II indicate two biological replicates.

	**LR-$^{12}C_{LG}$**	**LR-$^{12}C^{*}_{LG}$+*Ab***		**LR-$^{13}C_{LG}$**	**LR-$^{13}C_{LG}$+*Ab***	
	**I**	**I**	**II**	**I**	**I**	**II**
**Lignin content (%)**[a]	86.1 ± 0.0	42.8 ± 5.2	42.2 ± 11.3	85.6 ± 0.1	43.9 ± 7.5	66.1 ± 3.3
**Lignin subunits (%)**						
H	16.8 ± 0.6	24.8 ± 0.8	24.4 ± 3.8	17.9 ± 0.5	25 ± 0.3	21.6 ± 1.3
G	48 ± 0.0	42.4 ± 0.6	42.9 ± 1.8	56.3 ± 0.1	54.6 ± 0.0	54.3 ± 0.0
S	35.3 ± 0.5	32.8 ± 0.2	32.8 ± 2.0	25.8 ± 0.6	20.4 ± 0.3	24.1 ± 1.3
*t*coumAlc	0.6 ± 0.0	0.4 ± 0.0	0.4 ± 0.1	0.7 ± 0.0	0.3 ± 0.1	0.4 ± 0.1
*t*conAlc	23 ± 0.3	12.9 ± 0.9	14.2 ± 4.1	16.9 ± 0.9	6.7 ± 1.7	9.9 ± 1.4
*t*sinapAlc	19.9 ± 0.6	13.1 ± 0.4	14.1 ± 3.4	13 ± 0.9	5.9 ± 1.1	9.2 ± 1.4
S/G	0.7 ± 0.0	0.8 ± 0.0	0.8 ± 0.0	0.5 ± 0.0	0.4 ± 0.0	0.4 ± 0.0
tsinapAlc/tconifAlc	0.9 ± 0.0	1.0 ± 0.0	1.0 ± 0.1	0.8 ± 0.0	0.9 ± 0.1	0.9 ± 0.0
**Structural moieties (%)**						
unsub	7.4 ± 0.0	9.8 ± 0.1	9.3 ± 0.9	8 ± 0.2	10.5 ± 0.6	9.8 ± 0.3
Methyl	3.1 ± 0.1	3.8 ± 0.1	3.6 ± 0.3	3.5 ± 0.1	4.0 ± 0.3	4.0 ± 0.1
Vinyl	31.4 ± 0.8	41.6 ± 1.2	40.9 ± 5.6	45.3 ± 1.5	59.1 ± 1.6	51.8 ± 2.5
4-VP	13.9 ± 0.5	21.3 ± 0.7	20.9 ± 3.5	14.6 ± 0.5	21.4 ± 0.2	18.3 ± 1.1
4-VG	13.4 ± 0.3	15.3 ± 0.4	15.1 ± 1.6	27.1 ± 0.8	33.5 ± 1.1	29.5 ± 1.3
Cα-ox	5.0 ± 0.0	7.5 ± 0.1	7.2 ± 0.5	3.8 ± 0.1	5.3 ± 0.3	5.5 ± 0.2
diketones	1.0 ± 0.0	1.6 ± 0.1	1.5 ± 0.2	0.7 ± 0.0	1.2 ± 0.1	0.9 ± 0.0
ketones	0.2 ± 0	0.2 ± 0.0	0.2 ± 0.0	0.1 ± 0.0	0.1 ± 0.0	0.1 ± 0.0
Cβ-ox	1.5 ± 0	1.9 ± 0.1	1.9 ± 0.3	1.2 ± 0.0	1.6 ± 0.0	1.3 ± 0.0
Cγ-ox	47 ± 0.9	30.4 ± 1.5	32.5 ± 7.7	33.5 ± 1.8	15.8 ± 2.9	22.6 ± 2.9
misc	4.6 ± 0.1	5.0 ± 0.1	4.7 ± 0.0	4.6 ± 0.0	3.7 ± 0.1	4.9 ± 0.2
PhC_{γ}[b]	53.5 ± 0.9	38.1 ± 1.4	39.9 ± 7.3	39.2 ± 1.8	21.3 ± 2.9	28.9 ± 3.1
PhCγ-diketones[c]	52.6 ± 0.9	36.5 ± 1.5	38.4 ± 7.6	38.6 ± 1.8	20.1 ± 2.8	28 ± 3.1

[a] Lignin content (% w/w), dry matter based, and including H-phenol units.
[b] Pyrolysis products with intact α-, β-, γ-carbon chain.
[c] PhCγ excluding diketones.

Table S5

Lignin content, analyzed by py-GC-MS, and relative abundance of pyrolysis products of the water soluble lignin fractions (LS) corrected for RRF (*18*) See figure 1 for fractionation scheme and sample codes. Sum on the bases of previous structural classification (*18, 40, 55*). Average and standard deviation were derived from technical duplicates. I and II indicate two biological replicates.

	**LS-$^{12}C_{LG}$**	**LS-$^{12}C*_{LG}$+*Ab***		**LS-$^{13}C_{LG}$+*Ab***	**LS-$^{13}C_{LG}$+*Ab***	
	**I**	**I**	**II**	**I**	**I**	**II**
**Lignin content (%)[a]**	56.7 ± 7.4	10.2 ± 0.7	9.6 ± 1.2	52.6 ± 4.6	10.1 ± 0.2	9.6 ± 0.4
**Lignin subunits (%)**						
H	21 ± 1.3	25.5 ± 1.4	21.9 ± 0.3	12.3 ± 2.7	17.9 ± 4	21.8 ± 0.8
G	63.9 ± 0.7	58.3 ± 0.7	61.5 ± 0.1	52.3 ± 11.6	54.9 ± 5.4	60.8 ± 0.2
S	15.1 ± 0.6	16.3 ± 0.7	16.6 ± 0.4	35.4 ± 14.3	27.2 ± 9.4	17.4 ± 0.6
*t*coumAlc	0.0 ± 0.0	0.0 ± 0.0	0.0 ± 0.0	0.1 ± 0.0	0.0 ± 0.0	0.0 ± 0.0
*t*conAlc	4 ± 0.4	1.6 ± 0.1	1.6 ± 0.0	6.1 ± 1.5	1.3 ± 0.3	1.5 ± 0.0
*t*sinapAlc	2.2 ± 0.2	2.0 ± 0.1	2.2 ± 0.0	3.4 ± 0.9	1.9 ± 0.5	2.0 ± 0.0
S/G	0.2 ± 0.0	0.3 ± 0.0	0.3 ± 0.0	0.8 ± 0.4	0.5 ± 0.2	0.3 ± 0.0
tsinapAlc/tconifAlc	0.5 ± 0.0	1.2 ± 0.0	1.4 ± 0.0	0.6 ± 0.0	1.5 ± 0	1.3 ± 0.0
**Structural moieties (%)**						
unsub	14.1 ± 0.4	12.6 ± 0.7	11.4 ± 0.0	6.5 ± 1.4	9.6 ± 2.2	9.0 ± 0.3
Methyl	1.5 ± 0.0	4.9 ± 0.4	5.3 ± 0.1	2.4 ± 0.1	5.4 ± 1.1	7.1 ± 0.1
Vinyl	53.7 ± 2.1	50.2 ± 1.7	43.2 ± 0.3	54.4 ± 9.4	47.1 ± 5	42.1 ± 0.9
4-VP	17.2 ± 1.3	16.3 ± 0.9	12.4 ± 0.2	9.8 ± 2.2	8.9 ± 2.0	10.4 ± 0.5
4-VG	35.1 ± 2.2	32.6 ± 2.0	29.6 ± 0.4	25.2 ± 5.5	23.7 ± 5.1	29.6 ± 0.5
Cα-ox	7.6 ± 3.8	10.7 ± 3.2	17.1 ± 0.0	7.6 ± 1.6	11.4 ± 2.6	14.8 ± 0.5
diketones	4.6 ± 3.9	6.4 ± 3.6	12.2 ± 0.2	4.9 ± 1.0	8.0 ± 1.8	10.0 ± 0.5
ketones	0.5 ± 0.0	0.7 ± 0.1	0.8 ± 0.0	0.3 ± 0.1	0.5 ± 0.1	0.6 ± 0.0
Cβ-ox	2.0 ± 0.1	1.6 ± 0.4	2.0 ± 0.2	5.5 ± 1.0	1.1 ± 0.1	2.1 ± 0.3
Cγ-ox	9.0 ± 0.7	5.3 ± 0.3	5.6 ± 0.1	12.4 ± 3.1	4.6 ± 1.0	4.7 ± 0.5
misc	12.1 ± 0.4	14.7 ± 0.6	15.5 ± 0.2	11.2 ± 2.2	20.8 ± 1.9	20.2 ± 0.2
PhC_{γ}[b]	25.8 ± 2.6	27.3 ± 3.0	34.3 ± 0.3	28.6 ± 6.4	33.9 ± 0.6	35.7 ± 0.8
PhCγ-diketones[c]	21.2 ± 1.3	20.9 ± 0.6	22.1 ± 0.5	23.8 ± 5.4	25.9 ± 1.3	25.7 ± 0.3

[a] Lignin content (% w/w), dry matter based, and including H-phenol units.

[b] Pyrolysis products with intact α-, β-, γ-carbon chain.

[c] PhCγ excluding diketones.

Table S6

Lignin content, analyzed by py-GC-MS, and relative abundance of pyrolysis products of the lignin incorporated in the fungal biomass (FBM) corrected for RRF (*18*) See figure 1 for fractionation scheme and sample codes. Sum on the bases of previous structural classification (*18, 40, 55*). Average and standard deviation were derived from technical duplicates. I and II indicate two biological replicates.

	**FBM^{12}C*$_{LG}$+Ab**		**FBM^{13}C$_{LG}$+*Ab***	
	**I**	**II**	**I**	**II**
**Lignin content (%)**[a]	59.0 ± 0.3	58.7 ± 0.0	64.8 ± 0.2	60.2 ± 0.2
**Lignin subunits (%)**				
H	17.8 ± 0.2	17.6 ± 0.0	23.4 ± 0.3	23.4 ± 0.1
G	47.6 ± 0.1	47.5 ± 0.0	46.1 ± 0.0	45.8 ± 0.0
S	34.6 ± 0.1	34.9 ± 0.0	30.5 ± 0.3	30.9 ± 0.1
*t*coumAlc	0.6 ± 0.0	0.6 ± 0.0	1.0 ± 0.0	0.9 ± 0.0
*t*conAlc	22.1 ± 0.4	22 ± 0.1	22.3 ± 0.1	22.2 ± 0.1
*t*sinapAlc	18.9 ± 0.2	19.2 ± 0.0	16.6 ± 0.4	16.9 ± 0.2
S/G	0.7 ± 0.0	0.7 ± 0.0	0.7 ± 0.0	0.7 ± 0.0
tsinapAlc/tconifAlc	0.9 ± 0.0	0.9 ± 0.0	0.7 ± 0.0	0.8 ± 0.0
**Structural moieties (%)**				
unsub	7.8 ± 0.2	7.9 ± 0.0	6.7 ± 0.0	6.8 ± 0.0
Methyl	3.2 ± 0.0	3.2 ± 0.0	2.5 ± 0.0	2.4 ± 0.0
Vinyl	32.7 ± 0.4	32.4 ± 0.1	37.5 ± 0.4	37.3 ± 0.3
4-VP	14.6 ± 0.2	14.5 ± 0.0	22.1 ± 0.3	22.1 ± 0.1
4-VG	13.8 ± 0.3	13.7 ± 0.1	11.9 ± 0.1	11.6 ± 0.2
Cα-ox	5.0 ± 0.0	5.0 ± 0.0	4.6 ± 0.0	4.5 ± 0.0
diketones	1.0 ± 0.0	1.1 ± 0.0	1.0 ± 0.0	1.0 ± 0.0
ketones	0.2 ± 0.0	0.2 ± 0.0	0.2 ± 0.0	0.2 ± 0.0
Cβ-ox	1.6 ± 0.0	1.6 ± 0.0	1.5 ± 0.0	1.5 ± 0.0
Cγ-ox	45.2 ± 0.7	45.4 ± 0.0	42.7 ± 0.5	42.9 ± 0.4
misc	4.6 ± 0.0	4.5 ± 0.0	4.6 ± 0.0	4.6 ± 0.0
PhC_{γ}[b]	51.8 ± 0.7	51.9 ± 0.0	49.1 ± 0.5	49.4 ± 0.4
PhCγ-diketones[c]	50.7 ± 0.6	50.9 ± 0.0	48.1 ± 0.5	48.4 ± 0.4

[a] Lignin content (% w/w), dry matter based, and including H-phenol units.
[b] Pyrolysis products with intact α-, β-, γ-carbon chain.
[c] PhCγ excluding diketones.

Table S7

Semiquantitative ^{1}H-^{13}C HSQC NMR structural characterization of water soluble (LS) and insoluble (LR) residual lignin after fungal treatment of biological duplicates ($^{13}C_{Lg}$+*Ab*^{I}, $^{13}C_{Lg}$+*Ab*^{II}), compared with the 'control' lignin without addition of *Ab* ($^{13}C_{Lg}$). See Figure 1 for explanation of the sample codes.

	S			R		
	**$^{13}C_{Lg}$+*Ab*^{I}**	**$^{13}C_{Lg}$+*Ab*^{II}**	**$^{13}C_{Lg}$**	**$^{13}C_{Lg}$+*Ab*^{I}**	**$^{13}C_{Lg}$+*Ab*^{II}**	**$^{13}C_{Lg}$**
**Lignin subunits (%)**[a]						
H[b]	0.0	0.0	0.0	0.4	0.5	0.0
G	22.0	27.5	49.7	45.8	47.0	49.2
Gox	22.0	19.1	6.0	0.9	0.0	0.0
S	36.9	35.0	38.7	51.8	50.8	50.8
Sox	19.0	18.3	5.5	1.1	1.7	0.0
S/G	1.3	1.1	0.8	1.1	1.1	1.0
Total C_{α}-ox	41.1	37.5	11.5	2.0	1.7	0.0
**Hydroxycinnamates (per 100 ar)**[c]						
pCA	2.5	2.1	7.6	19.4	18.4	19.5
FA	9.8	8.3	13.5	0.0	0.0	0.0
**Flavonoids (per 100 ar)**[c]						
Tricin	4.6	4.3	5.5	9.9	7.9	6.9
**Lignin interunit linkages (per 100 ar)**[c,d]						
β-*O*-4 G (β)	12 (47)	12 (46)	13 (39)	11 (32)	10 (30)	14 (37)
β-*O*-4 S (β)	7 (27)	8 (30)	11 (32)	14 (42)	14 (41)	15 (38)
β-*O*-4 C_{α}-ox	0 (0)	0 (0)	0 (0)	2 (5)	2 (4)	0 (0)
β-*O*-4 (electron withdrawing)	5 (20)	5 (19)	5 (14)	3 (11)	5 (14)	6 (15)
Total β-*O*-4[e]	25 (94)	24 (95)	28 (84)	30 (89)	31 (89)	35 (90)
Phenylcoumaran	2 (6)	1 (5)	4 (13)	3 (8)	3 (8)	3 (7)
Resinol	0 (0)	0 (0)	1 (3)	1 (3)	1 (3)	1 (3)
sum	26 (100)	25 (100)	33 (100)	33 (100)	35 (100)	39 (100)
**End units (per 100 ar)**[c]						
Cinnamyl alcohol	0.0	0.0	1.8	1.4	1.3	1.3
Cinnamaldehyde	1.1	0.9	0.9	0.4	0.7	0.0
Benzaldehyde	7.3	8.4	3.7	1.0	1.2	0.0
HPV/HPS	13.6	14.0	0.0	0.1	0.1	0.0
DHPV/DHPS	0.4	0.4	0.0	0.0	0.0	0.0
**Erythro/threo**[f]	3.0	3.0	1.9	1.7	1.7	1.8

[a] Relative distribution of lignin subunits ($H+G+G_{ox}+S+S_{ox}=100$)

[b] $H_{2,6}$ integrals corrected for phenylalanine peak ($PHE_{3,5}$)

[c] Relative distribution of total interunit linkages in parenthesis

[d] Relative volume integral of substructure versus volume integral of ($H+G+G_{ox}+S+S_{ox}$)

[e] Absolute and relative in brackets of total β-*O*-4 linkages based on β signal

[f] Ratio of $A_{\beta}(S/G\text{-}S)_{erythro}$ and $A_{\beta}(S/G\text{-}S)_{threo}$; diastereomers for β-*O*-4′ aryl ethers coupled to G-units were not resolved.

Table S8

Fatty acid composition and content (based on dry matter) of *Agaricus bisporus* fungal biomass cultivated on different substrates (see Figure 1 for codes), and fatty acid content and composition of the ^{13}C lignin isolate ($^{13}C_{LG}$).

Composition (mol (%))	^{13}CG+*Ab*	$^{12}C_{AX}$+*Ab*	$^{13}C_{LG}$+*Ab*	$^{12}C^*_{LG}$+*Ab*	$^{13}C_{LG}$
**C12:0**	0.0 ± 0.0	0.0 ± 0.0	0.6 ± 0.6	0.3 ± 0.3	0.0 ± 0.0
**C13:0**	0.0 ± 0.0	0.0 ± 0.0	0.3 ± 0.3	0.0 ± 0.0	0.0 ± 0.0
**C14:0**	0.0 ± 0.0	0.0 ± 0.0	2.5 ± 1.2	1.7 ± 0.3	0.0 ± 0.0
**C15:0**	1.1 ± 0.6	1.7 ± 0.2	3.6 ± 0.0	4.7 ± 1.8	0.0 ± 0.0
**C16:0**	13.2 ± 0.1	11.6 ± 0.1	17.3 ± 0.0	18.4 ± 1.8	42.2 ± 1.6
**C16:1, *c***	0.0 ± 0.0	0.0 ± 0.0	0.0 ± 0.0	0.0 ± 0.0	0.0 ± 0.0
**C17:0**	2.6 ± 0.0	1.9 ± 0.1	2.8 ± 0.4	2.2 ± 0.0	0.0 ± 0.0
**C18:0**	6.5 ± 0.2	6.0 ± 0.2	12.0 ± 1.0	12.2 ± 2.0	14.6 ± 1.5
**C18:1, *t/c* 9 mix**	6.7 ± 1.0	4.7 ± 0.0	6.9 ± 2.0	5.7 ± 0.9	19.9 ± 11.1
**C18:2, *c* 9,12**	62.9 ± 2.3	66.4 ± 0.8	43.8 ± 3.1	47.8 ± 2.6	0.0 ± 0.0
**C20:0**	3.8 ± 1.0	4.1 ± 0.8	4.3 ± 1.4	2.2 ± 0.1	23.3 ± 8
**C20:1, *c* 11**	0.0 ± 0.0	0.0 ± 0.0	0.0 ± 0.0	0.0 ± 0.0	0.0 ± 0.0
**C22:0**	3.2 ± 0.1	3.7 ± 0.6	6.0 ± 1.1	4.9 ± 0.3	0.0 ± 0.0
**Fatty acid content (% (w/w))**	7.7 ± 0.6	6.4 ± 1.5	1.7 ± 0	3.2 ± 0.4	0.6 ± 0.2

Table S9

Fatty acid methyl esters, ergosterol and selected fragments with chemical formulas and exact masses. The screened *m/z* of ^{13}C isotopomers of the selected fragments (F) or parent (P) were used for integration to determine the ^{13}C fractional enrichment with mass precision of 10 ppm.

Compound	Code	Chem. formula	RT $_{(min)}$	Exact mass	Screened *m/z* ^{13}C isotopomers
**Methyl pentadecanoate**	**C15:0**	**$C_{16}H_{32}O_2$**	**6.91**	**256.24023**	
Parent	C15:0	$C_{16}H_{32}O_2$	6.91	256.24023	257.24359; 258.24694; 259.25029; 260.25365; 261.25700; 262.26036; 263.26371; 264.26707; 265.27042; 266.27378; 267.27713; 268.28049; 269.28384; 270.28720; 271.29055; 272.29391;
Fragment 1 (F1)	C15:0-F1	$C_3H_6O_2$	6.91	74.03678	74.03625; 75.03957; 76.04294; 77.0462
**Methyl palmitate**	**C16:0**	**$C_{17}H_{34}O_2$**	**8.69**	**270.25588**	
Fragment 1 (F1)	C16:0-F1	$C_{15}H_{29}O_2$	8.69	241.21676	242.22011; 243.22346; 244.22682; 245.23017; 246.23353; 247.23688; 248.24024; 249.24359; 250.24695; 251.25030; 252.25366; 253.25701; 254.26037; 255.26372; 256.26708;
Fragment 2 (F2)	C16:0-F2	$C_4H_7O_2$	8.69	87.04460	87.04395; 88.04729; 89.05067; 90.054; 91.05744
**Methyl heptadecanoate**	**C17:0**	**$C_{18}H_{36}O_2$**	**10.6**	**284.27153**	
Fragment 1 (F1)	C17:0-F1	$C_{16}H_{31}O_2$	10.6	255.23241	256.23576; 257.23911; 258.24247; 259.24582; 260.24918; 261.25253; 262.25589; 263.25924; 264.26260; 265.26595; 266.26931; 267.27266; 268.27602; 269.27937; 270.28273; 271.28608;
Fragment 2 (F2)	C17:0-F2	$C_4H_7O_2$	10.6	87.04460	87.04460; 88.04796; 89.05131; 90.05467; 91.05802
**Methyl stearate**	**C18:0**	**$C_{19}H_{38}O_2$**	**12.6**	**298.28718**	
Fragment 1 (F1)	C18:0-F1	$C_{16}H_{31}O_2$	12.6	255.23241	256.23576; 257.23911; 258.24247; 259.24582; 260.24918; 261.25253; 262.25589; 263.25924; 264.26260; 265.26595; 266.26931; 267.27266; 268.27602; 269.27937; 270.28273; 271.28608;
Fragment 2 (F2)	C18:0-F2	$C_4H_7O_2$	12.6	87.04460	87.04460; 88.04796; 89.05131; 90.05467; 91.05802
**Methyl oleate**	**C18:1**	**$C_{19}H_{36}O_2$**	**12.89**	**296.27153**	
Fragment 1 (F1)	C18:1-F1	$C_{18}H_{32}O$	12.89	264.24532	265.24867; 266.25203; 267.25538; 268.25873; 269.26209; 270.26544; 271.26880; 272.27215; 273.27551; 274.27886; 275.28222; 276.28557; 277.28893; 278.29228; 279.29564; 280.29899; 281.30235; 282.30570;

Fragment 2 (F2)	C18:1-F2	C_6H_9	12.89	81.07043	81.06985; 82.0732; 83.07653; 84.07989; 85.08324; 86.08659; 87.08997
**Methyl linoleate**	**C18:2**	$\mathbf{C_{19}H_{34}O_2}$	**13.7**	**294.25588**	
Fragment 1 (F1)	C18:2-F1	$C_{18}H_{30}O$	13.7	262.22967	263.23302; 264.23638; 265.23973; 266.24308; 267.24644; 268.24979; 269.25315; 270.25650; 271.25986; 272.26321; 273.26657; 274.26992; 275.27328; 276.27663; 277.27999; 278.28334; 279.28670; 280.29005
Fragment 2 (F2)	C18:2-F2	C_5H_7	13.7	67.05477	67.05417; 68.05754; 69.06087 70.06425; 71.06758; 72.07092
**Methyl behenate**	**C22:0**	$\mathbf{C_{23}H_{46}O_2}$	**20.6**	**354.34978**	
Parent	C22:0	$C_{23}H_{46}O_2$	20.6	354.34978	355.35314; 356.35649; 357.35985; 358.36320; 359.36655; 360.36991; 361.37326; 362.37662; 363.37997; 364.38333; 365.38668; 366.39004; 367.39339; 368.39675; 369.40010; 370.40346; 371.40681; 372.41017; 373.41352; 374.41688; 375.42023; 376.42359; 377.42694;
Fragment 1 (F1)		$C_4H_7O_2$	20.6	87.04460	87.04400; 88.04736; 89.05078; 90.05404; 91.05743
**Ergosterol**	**E**	$\mathbf{C_{28}H_{44}O}$	**52.1**	**396.33922**	396.33922; 397.34257; 398.34593; 399.34928; 400.35264; 401.35599 402.35935; 403.36270; 404.36605; 405.36941; 406.37276; 407.37612 408.37947; 409.38283; 410.38618; 411.38954; 412.39289; 413.39625 414.39960; 415.40296; 416.40631; 417.40967; 418.41302; 419.41638 420.41973; 421.42309; 422.42644; 423.42980; 424.43315

Table S10

^{13}C fractional labelling of fungal biomass compounds. Fatty acids, carbohydrates, amino acids, of *A. bisporus* (*Ab*) fungal biomass from $^{12}C_G$+*Ab*, $^{12}C_{Lg}$+*Ab*, $^{13}C_G$+*Ab*, $^{12}C^*_{Lg}$+*Ab*, and $^{13}C_{Lg}$+*Ab*. All results are the average of biological duplicates, and error bars represent the standard deviations.

		$^{12}C_G$+*Ab*	$^{12}C_{Lg}$+*Ab*	$^{13}C_G$+*Ab*	$^{12}C^*_{Lg}$+*Ab*	$^{13}C_{Lg}$+*Ab*
**Alanine**	**A**	1.5 ± 0.0	1.8 ± 0.3	61.4 ± 1.3	11.1 ± 0.3	26.3 ± 2.5
**Glycine**	**G**	1.8 ± 0.1	2.1 ± 0.2	49.5 ± 0.7	12 ± 0.1	22.7 ± 1.7
**Valine**	**V**	1.5 ± 0.0	2.7 ± 0.3	55.1 ± 0.8	6.1 ± 0.6	24.1 ± 2.0
**Leucine**	**L**	1.9 ± 0.1	2.4 ± 0.3	43.5 ± 1.4	4.6 ± 0.1	25.2 ± 2.1
**Isoleucine**	**I**	2.1 ± 0.2	4.2 ± 0.8	46.2 ± 0.9	7.2 ± 0.3	22.4 ± 2.3
**Proline**	**P**	1.7 ± 0.1	2.5 ± 0.2	39.4 ± 1.0	4.0± 0.0	19.6 ± 2.7
**Methionine**	**M**	2 ± 0.1	2.8 ± 0.6	52.0 ± 0.5	6.3 ± 0.0	18.7 ± 1.6
**Serine**	**S**	1.4 ± 0.0	1.5 ± 0.0	51.6 ± 0.8	12.0 ± 0.0	19.1 ± 1.5
**Threonine**	**T**	1.3 ± 0.0	2.0 ± 0.1	47.9 ± 0.6	4.4 ± 0.0	14.9 ± 1.5
**Phenylalanine**	**F**	2.7 ± 0.5	1.8 ± 0.3	53.7 ± 0.1	5.1 ± 0.0	20.7 ± 1.5
**Aspartate**	**N**	1.5 ± 0.1	1.8 ± 0.1	48.9 ± 1.1	5.9 ± 0.1	17.5 ± 0.7
**Glutamate**	**Q**	1.7 ± 0.1	1.8 ± 0.0	47.7 ± 2.1	5.9 ± 0.0	15.9 ± 1.3
**Lysine**	**K**	1.9 ± 0.2	1.5 ± 0.4	40.7 ± 1.6	3.6 ± 0.1	10.5 ± 1.1
**Arginine**	**R**	2.7 ± 0.1	7.7 ± 0.3	44.6 ± 0.8	11.3 ± 1.0	14.6 ± 2.5
**Tyrosine**	**Y**	2.2 ± 0.5	5.1 ± 1.8	58.6 ± 0.7	9.3 ± 0.2	17.3 ± 1.6
**Ribose**	**R1**	1.1 ± 0.1	1.6 ± 0.4	73.6 ± 0.8	12.0 ± 0.4	12.5 ± 0.0
	**R2**	1.2 ± 0.0	2.1 ± 0.3	72.3 ± 0.7	11.9 ± 0.5	11.1 ± 1.0
**Glucose**	**G1**	2.1 ± 0.2	1.1 ± 0.0	73.4 ± 0.8	11.6 ± 0.0	11.1 ± 0.1
	**G1-2**	2.4 ± 0.1	1.9 ± 0.2	70.3 ± 0.9	10.9 ± 0.1	9.9 ± 0.8
	**G2**	2.4 ± 0.1	5.3 ± 1.6	71.2 ± 0.8	11.4 ± 0.3	11.7 ± 0.3
	**G2-2**	1.9 ± 0.2	1.4 ± 0.0	71.3 ± 0.9	10.2 ± 0.1	9.4 ± 0.7
**Glucosamine**	**GN1-1**	1.1 ± 0.0	1.2 ± 0.3	61.0 ± 0.2	6.4 ± 0.2	3.3 ± 0.3
	**GN1-2**	2.1 ± 0.4	5.7 ± 0.3	68.6 ± 0.3	9.3 ± 0.4	5.5 ± 0.2
	**GN2-1**	1.0 ± 0.0	1.0 ± 0.0	60.6 ± 0.5	7.5 ± 0.8	5.7 ± 0.1
	**GN2-2**	1.1 ± 0.2	1.1 ± 0.1	59.8 ± 0.3	7.0 ± 0.2	3.6 ± 0.1
**C15:0**	**P**	2.2 ± 0.6	1.3 ± 0.3	19.6 ± 10.6	0.5 ± 0.2	2.0 ± 1.6
	**F1**	0.4 ± 0.1	0.3 ± 0.0	32.5 ± 1.7	9.2 ± 1.0	18.7 ± 2.4
**C16:0**	**F1**	0.4 ± 0.0	0.2 ± 0.0	24.4 ± 3.6	16.4 ± 0.1	9.6 ± 2.1
	**F2**	0.4 ± 0.0	0.4 ± 0.1	27.1 ± 3.6	16.0 ± 0.1	11.0 ± 1.9
**C17:0**	**F1**	0.2 ± 0.1	0.2 ± 0.1	27.1 ± 0.7	9.6 ± 1.4	1.8 ± 0.4
	**F2**	0.3 ± 0.0	0.2 ± 0.0	31.4 ± 0.7	9.9 ± 1.1	7.0 ± 0.3
**C18:0**	**F1**	0.4 ± 0.0	0.1 ± 0.0	17.0 ± 4.4	4.7 ± 0.4	1.8 ± 0.2
	**F2**	0.3 ± 0.1	0.1 ± 0.0	17.9 ± 4.3	3.8 ± 0.2	2.4 ± 0.2
**C18:1**	**F1**	0.0 ± 0.0	0.0 ± 0.0	4.1 ± 2.6	0.0 ± 0.0	0.1 ± 0.0
	**F2**	0.2 ± 0	0.1 ± 0.0	14.0 ± 4.7	0.4 ± 0.1	0.3 ± 0.0
**C18:2**	**F1**	0.0 ± 0.0	0.1 ± 0.0	41.9 ± 1.6	2.8 ± 0.2	1.6 ± 0.1
	**F2**	0.4 ± 0.0	0.5 ± 0.0	35.6 ± 1.0	3.9 ± 0.3	6.6 ± 0.2
**C22:0**	**F1**	0.3 ± 0.2	0.1 ± 0.0	9.7 ± 1.5	1.0 ± 0.0	31.5 ± 2.8
	**F2**	0.4 ± 0.0	0.2 ± 0.0	28.9 ± 2.6	2.0 ± 0.1	31.1 ± 2.2

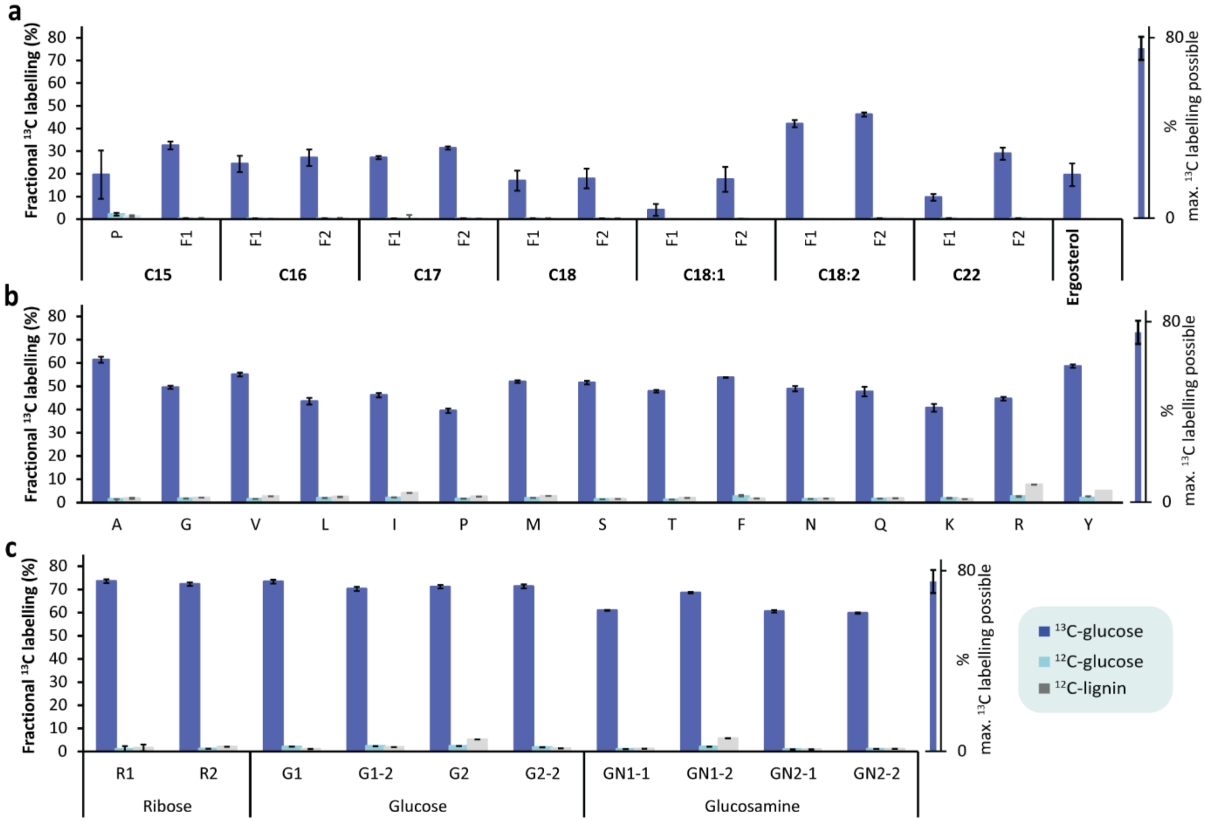

Fig. S7

^{13}C fractional labelling of fungal biomass compounds. Fatty acids and ergosterol (a), amino acids (b), carbohydrates (c), of *A. bisporus (Ab)* fungal biomass from $^{12}C_G$+*Ab* (light blue bars; glucose used as carbon source), from $^{13}C_G$+*Ab* (dark blue bars; ^{13}C-glucose used as carbon source), from $^{12}C_{Lg}$+*Ab* (grey bars; ^{12}C-lignin used as carbon source). All results are the average of biological duplicates, and error bars represent the standard deviations. The graphs on the right side indicate maximal labelling possible, calculated from fungal biomass increase and mycelium seed used, for $^{13}C_G$+*Ab* (dark blue).

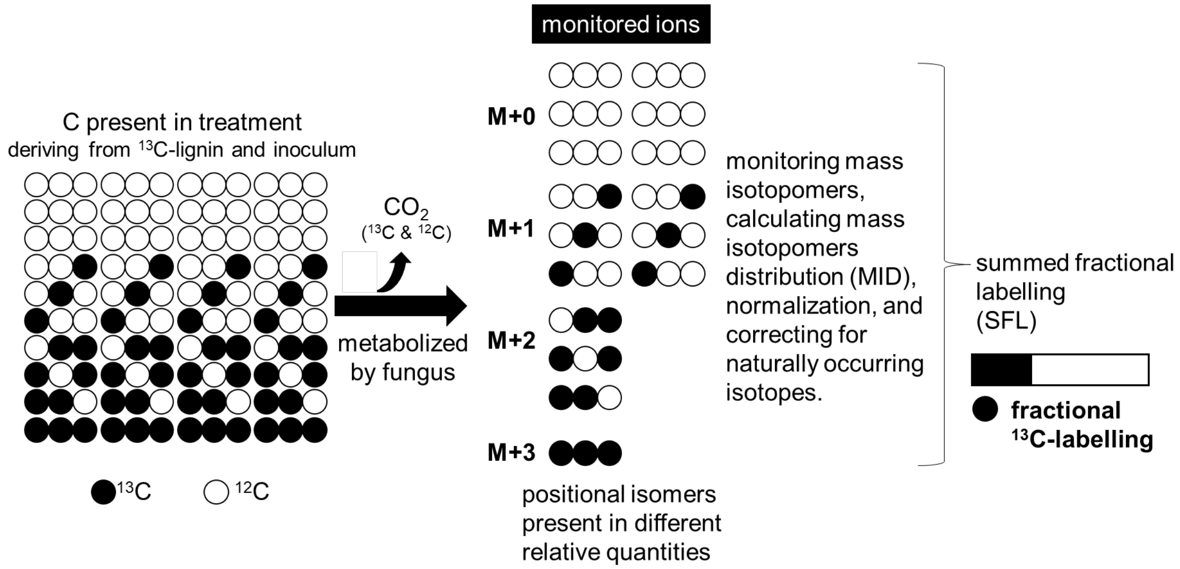

Fig. S8

Schematic explanation ^{13}C fractional labelling of fungal biomass compounds. C present in the treatment consist of ^{12}C (fungal inoculum and debris from inoculum) and ^{13}C (naturally occurring, and ^{13}C-lignin substrate). Fungus metabolizes C to CO_2 (extracellular and intracellular). Intracellularly, the C can further be converted and anabolized to new compounds (e.g., three carbon compounds). These newly formed compounds have are different mass isotopomers. The ^{13}C carbon can be implemented in the *de novo* biosynthesized compounds at different positions (positional isotopomers). Abundance of individual mass isotopomers is screened. Normalization of mass isotopomers abundances result in mass isotopomer distribution (MID). Further calculation steps involve the correction of the natural abundance of ^{13}C and then the summed fractional labelling can be calculated with the correction matrix. These calculations are explicitly explained in literature (*42, 43, 44, 56*).

.

Data S1. (separate file)

See excel file: SupplementarySecretome_AgaBisH97

REFERENCES

1. C . Liang, J. P. Schimel, J. D. Jastrow, The importance of anabolism in microbial control over soil carbon storage. *Nat. Microbiol.* **2**, 17105 (2017).

2. F. Tao, Y. Huang, B. A. Hungate, S. Manzoni, S. D. Frey, M. W. I. Schmidt, M. Reichstein, N. Carvalhais, P. Ciais, L. Jiang, J. Lehmann, Y. P. Wang, B. Z. Houlton, B. Ahrens, U. Mishra, G. Hugelius, T. D. Hocking, X. Lu, Z. Shi, K. Viatkin, R. Vargas, Y. Yigini, C. Omuto, A. A. Malik, G. Peralta, R. Cuevas-Corona, L. E. Di Paolo, I. Luotto, C. Liao, Y. S. Liang, V. S. Saynes, X. Huang, Y. Luo, Microbial carbon use efficiency promotes global soil carbon storage. *Nature* **618**, 981–985 (2023).

3. X. Kang, A. Kirui, M. C. Dickwella Widanage, F. Mentink-Vigier, D. J. Cosgrove, T. Wang, Lignin-polysaccharide interactions in plant secondary cell walls revealed by solid-state NMR. *Nat. Commun.* **10**, 347 (2019).

4. S. M. Cragg, G. T. Beckham, N. C. Bruce, T. D. H. Bugg, D. L. Distel, P. Dupree, A. G. Etxabe, B. S. Goodell, J. Jellison, J. E. McGeehan, S. J. McQueen-Mason, K. Schnorr, P. H. Walton, J. E. M. Watts, M. Zimmer, Lignocellulose degradation mechanisms across the tree of life. *Curr. Opin. Chem. Biol.* **29**, 108–119 (2015).

5. T . Kijpornyongpan, A. Schwartz, A. Yaguchi, D. Salvachúa, Systems biology-guided understanding of white-rot fungi for biotechnological applications: A review. *iScience* **25**, 104640 (2022).

6. R. Vanholme, B. Demedts, K. Morreel, J. Ralph, W. Boerjan, Lignin biosynthesis and structure. *Plant Physiol.* **153**, 895–905 (2010).

7. D. L. Moorhead, G. Lashermes, R. L. Sinsabaugh, M. N. Weintraub, Calculating co-metabolic costs of lignin decay and their impacts on carbon use efficiency. *Soil Biol. Biochem.* **66**, 17–19 (2013).

8. T . Klotzbücher, K. Kaiser, G. Guggenberger, C. Gatzek, K. Kalbitz, A new conceptual model for the fate of lignin in decomposing plant litter. *Ecology* **92**, 1052–1062 (2011).

9. J. M. Talbot, K. K. Treseder, Interactions among lignin, cellulose, and nitrogen drive litter chemistry-decay relationships. *Ecology* **93**, 345–354 (2012).

10. C . del Cerro, E. Erickson, T. Dong, A. R. Wong, E. K. Eder, S. O. Purvine, H. D. Mitchell, K. K. Weitz, L. M. Markillie, M. C. Burnet, D. W. Hoyt, R. K. Chu, J. F. Cheng, K. J. Ramirez, R. Katahira, W. Xiong, M. E. Himmel, V. Subramanian, J. G. Linger, D. Salvachúa,

Intracellular pathways for lignin catabolism in white-rot fungi. *Proc. Natl. Acad. Sci. U.S.A.* **118**, e2017381118 (2021).

11. E . Morin, A. Kohler, A. R. Baker, M. Foulongne-Oriol, V. Lombard, L. G. Nagy, R. A. Ohm, A. Patyshakuliyeva, A. Brun, A. L. Aerts, A. M. Bailey, C. Billette, P. M. Coutinho, G. Deakin, H. Doddapaneni, D. Floudas, J. Grimwood, K. Hildén, U. Kües, K. M. Labutti, A. Lapidus, E. A. Lindquist, S. M. Lucas, C. Murat, R. W. Riley, A. A. Salamov, J. Schmutz, V. Subramanian, H. A. B. Wösten, J. Xu, D. C. Eastwood, G. D. Foster, A. S. M. Sonnenberg, D. Cullen, R. P. de Vries, T. Lundell, D. S. Hibbett, B. Henrissat, K. S. Burton, R. W. Kerrigan, M. P. Challen, I. V. Grigoriev, F. Martin, Genome sequence of the button mushroom *Agaricus bisporus* reveals mechanisms governing adaptation to a humic-rich ecological niche. *Proc. Natl. Acad. Sci. U.S.A.* **109**, 17501–17506 (2012).
12. C . H. Vane, G. D. Abbott, I. M. Head, The effect of fungal decay (*Agaricus bisporus*) on wheat straw lignin using pyrolysis–GC–MS in the presence of tetramethylammonium hydroxide (TMAH). *J. Anal. Appl. Pyrolysis* **60**, 69–78 (2001).
13. E . Jurak, A. M. Punt, W. Arts, M. A. Kabel, H. Gruppen, Fate of carbohydrates and lignin during composting and mycelium growth of *Agaricus bisporus* on wheat straw based compost. *PLOS ONE* **10**, e0138909 (2015).
14. K. Iiyama, B. A. Stone, B. J. Macauley, Compositional changes in compost during composting and growth of *Agaricus bisporus*. *Appl. Environ. Microbiol.* **60**, 1538–1546 (1994).
15. K. Duran, J. Miebach, G. van Erven, J. J. P. Baars, R. N. J. Comans, T. W. Kuyper, M. A. Kabel, Oxidation-driven lignin removal by *Agaricus bisporus* from wheat straw-based compost at industrial scale. *Int. J. Biol. Macromol.* **246**, 125575 (2023).
16. A. J. Durrant, D. A. Wood, R. B. Cain, Lignocellulose biodegradation by *Agaricus bisporus* during solid substrate fermentation. *J. Gen. Microbiol.* **137**, 751–755 (1991).
17. R. Ten Have, H. Wijngaard, N. A. E. Ariës-Kronenburg, G. Straatsma, P. J. Schaap, Lignin degradation by (*Agaricus bisporus*) accounts for a 30% increase in bioavailable holocellulose during cultivation on compost. *J. Agric. Food Chem.* **51**, 2242–2245 (2003).
18. G. van Erven, R. De Visser, D. W. H. Merkx, W. Strolenberg, P. De Gijsel, H. Gruppen, M. A. Kabel, Quantification of lignin and its structural features in plant biomass

Using13C lignin as internal standard for pyrolysis-GC-SIM-MS. *Anal. Chem.* **89**, 10907–10916 (2017).

19. A. Rodriguez, D. Salvachúa, R. Katahira, B. A. Black, N. S. Cleveland, M. Reed, H. Smith, E. E. K. Baidoo, J. D. Keasling, B. A. Simmons, G. T. Beckham, J. M. Gladden, Base-catalyzed depolymerization of solid lignin-rich streams enables microbial conversion. *ACS Sustain. Chem. Eng.* **5**, 8171–8180 (2017).
20. E . Drula, M. L. Garron, S. Dogan, V. Lombard, B. Henrissat, N. Terrapon, The carbohydrate-active enzyme database: Functions and literature. *Nucleic Acids Res.* **50**, D571–D577 (2022).
21. A. Patyshakuliyeva, H. Post, M. Zhou, E. Jurak, A. J. R. Heck, K. S. Hildén, M. A. Kabel, M. R. Mäkelä, M. A. F. Altelaar, R. P. Vries, Uncovering the abilities of *Agaricus bisporus* to degrade plant biomass throughout its life cycle. *Environ. Microbiol.* **17**, 3098–3109 (2015).
22. F. J. Ruiz-Dueñas, Á. T. Martínez, Microbial degradation of lignin: How a bulky recalcitrant polymer is efficiently recycled in nature and how we can take advantage of this. *Microb. Biotechnol.* **2**, 164–177 (2009).
23. K. Hildén, M. R. Mäkelä, P. Lankinen, T. Lundell, *Agaricus bisporus* and related Agaricus species on lignocellulose: Production of manganese peroxidase and multicopper oxidases. *Fungal Genet. Biol.* **55**, 32–41 (2013).
24. K. Duran, J. Magnin, A. H. P. America, M. Peng, R. Hilgers, R. P. de Vries, J. J. P. Baars, W. J. H. van Berkel, T. W. Kuyper, M. A. Kabel, The secretome of Agaricus bisporus: Temporal dynamics of plant polysaccharides and lignin degradation. *iScience* **26**, 107087 (2023).
25. T . Li, H. Liang, B. Wu, D. Lan, Y. Ma, F. Hollmann, Y. Wang, A novel unspecific peroxygenase from *Agaricus bisporus var. bisporus* for biocatalytic oxyfunctionalisation reactions. *Mol. Catal.* **546**, 113275 (2023).
26. M. Hofrichter, R. Ullrich, M. J. Pecyna, C. Liers, T. Lundell, New and classic families of secreted fungal heme peroxidases. *Appl. Microbiol. Biotechnol.* **87**, 871–897 (2010).
27. M. Kinne, M. Poraj-Kobielska, R. Ullrich, P. Nousiainen, J. Sipilä, K. Scheibner, K. E. Hammel, M. Hofrichter, Oxidative cleavage of non-phenolic β-*O*-4 lignin model dimers by an extracellular aromatic peroxygenase. *Holzforschung* **65**, 673–679 (2011).
28. B. Berg, H. Staaf, Decomposition rate and chemical changes of scots pine needle litter. I. Influence of stand age. *Ecological Bulletins*, 363–372 (1980).

29. R. J. M. Lubbers, A. Dilokpimol, J. Visser, M. R. Mäkelä, K. S. Hildén, R. P. de Vries, A comparison between the homocyclic aromatic metabolic pathways from plant-derived compounds by bacteria and fungi. *Biotechnol. Adv.* **37**, 107396 (2019).
30. C . S. Harwood, R. E. Parales, The β-ketoadipate pathway and the biology of self-identity. *Annu. Rev. Microbiol.* **50**, 553–590 (1996).
31. Z. Holesova, M. Jakubkova, I. Zavadiakova, I. Zeman, L. Tomaska, J. Nosek, Gentisate and 3-oxoadipate pathways in the yeast candida parapsilosis: Identification and functional analysis of the genes coding for 3-hydroxybenzoate 6-hydroxylase and 4-hydroxybenzoate 1-hydroxylase. *Microbiology* **157**, 2152–2163 (2011).
32. A. H. Westphal, D. Tischler, W. J. H. van Berkel, Natural diversity of FAD-dependent 4-hydroxybenzoate hydroxylases. *Arch. Biochem. Biophys.* **702**, 108820 (2021).
33. M. Kanehisa, S. Goto, KEGG: Kyoto Encyclopedia of Genes and Genomes. *Nucleic Acids Res.* **28**, 27–30 (2000).
34. M. Kanehisa, M. Furumichi, Y. Sato, M. Kawashima, M. Ishiguro-Watanabe, KEGG for taxonomy-based analysis of pathways and genomes. *Nucleic Acids Res.* **51**, D587–D592 (2023).
35. M. S. Patel, T. E. Roche, Molecular biology and biochemistry of pyruvate dehydrogenase complexes 1. *FASEB J.* **4**, 3224–3233 (1990).
36. G. T. Beckham, C. W. Johnson, E. M. Karp, D. Salvachúa, D. R. Vardon, Opportunities and challenges in biological lignin valorization. *Curr. Opin. Biotechnol.* **42**, 40–53 (2016).
37. J. Becker, C. Wittmann, A field of dreams: Lignin valorization into chemicals, materials, fuels, and health-care products. [Preprint] (2019). https://doi.org/10.1016/j. biotechadv.2019.02.016.
38. M. A. Kabel, E. Jurak, M. R. Mäkelä, R. P. de Vries, Occurrence and function of enzymes for lignocellulose degradation in commercial *Agaricus bisporus* cultivation. *Appl. Microbiol. Biotechnol.* **101**, 4363–4369 (2017).
39. G. van Erven, R. De Visser, P. De Waard, W. J. H. Van Berkel, M. A. Kabel, Uniformly 13C labeled lignin internal standards for quantitative pyrolysis-GC-MS analysis of grass and wood. *ACS Sustain. Chem. Eng.* **7**, 20070–20076 (2019).
40. J. C. Del Río, J. Rencoret, P. Prinsen, Á. T. Martínez, J. Ralph, A. Gutiérrez, Structural characterization of wheat straw lignin as revealed by analytical pyrolysis, 2D-NMR, and reductive cleavage methods. *J. Agric. Food Chem.* **60**, 5922–5935 (2012).

41. G. A. de Ruiter, H. A. Schols, A. G. J. Voragen, F. M. Rombouts, Carbohydrate analysis of water-soluble uronic acid-containing polysaccharides with high-performance anion-exchange chromatography using methanolysis combined with TFA hydrolysis is superior to four other methods. *Anal. Biochem.* **207**, 176–185 (1992).

42. M. Kohlstedt, C. Wittmann, GC-MS-based 13C metabolic flux analysis resolves the parallel and cyclic glucose metabolism of *Pseudomonas putida* KT2440 and *Pseudomonas aeruginosa* PAO1. *Metab. Eng.* **54**, 35–53 (2019).

43. W. A. van Winden, C. Wittmann, E. Heinzle, J. J. Heijnen, Correcting mass isotopomer distributions for naturally occurring isotopes. *Biotechnol. Bioeng.* **80**, 477–479 (2002).

44. T . Christiansen, B. Christensen, J. Nielsen, Metabolic network analysis of *Bacillus clausii* on minimal and semirich medium using 13C-labeled glucose. *Metab. Eng.* **4**, 159–169 (2002).

45. E . D. Dodds, M. R. McCoy, L. D. Rea, J. M. Kennish, Gas chromatographic quantification of fatty acid methyl esters: Flame ionization detection vs. electron impact mass spectrometry. *Lipids* **40**, 419–428 (2005).

46. J. Pi, X. Wu, Y. Feng, Fragmentation patterns of five types of phospholipids by ultra-high-performance liquid chromatography electrospray ionization quadrupole time-of-flight tandem mass spectrometry. *Anal. Methods* **8**, 1319–1332 (2016).

47. A. Patyshakuliyeva, E. Jurak, A. Kohler, A. Baker, E. Battaglia, W. Bruijn, K. S. Burton, M. P. Challen, P. M. Coutinho, D. C. Eastwood, B. S. Gruben, M. R. Mäkelä, F. Martin, M. Nadal, J. van den Brink, A. Wiebenga, M. Zhou, B. Henrissat, M. Kabel, H. Gruppen, R. P. Vries, Carbohydrate utilization and metabolism is highly differentiated in *Agaricus bisporus*. *BMC Genomics* **14**, 663 (2013).

48. Y. Perez-Riverol, J. Bai, C. Bandla, D. García-Seisdedos, S. Hewapathirana, S. Kamatchinathan, D. J. Kundu, A. Prakash, A. Frericks-Zipper, M. Eisenacher, M. Walzer, S. Wang, A. Brazma, J. A. Vizcaíno, The PRIDE database resources in 2022: A hub for mass spectrometry-based proteomics evidences. *Nucleic Acids Res.* **50**, D543–D552 (2022).

49. J. C. Del Río, A. G. Lino, J. L. Colodette, C. F. Lima, A. Gutiérrez, Á. T. Martínez, F. Lu, J. Ralph, J. Rencoret, Differences in the chemical structure of the lignins from sugarcane bagasse and straw. *Biomass Bioenergy* **81**, 322–338 (2015).

50. J. Zeng, G. L. Helms, X. Gao, S. Chen, Quantification of wheat straw lignin structure by comprehensive NMR analysis. *J. Agric. Food Chem.* **61**, 10848–10857 (2013).

51. G. van Erven, R. Hilgers, P. de Waard, E. J. Gladbeek, W. J. H. Van Berkel, M. A. Kabel, Elucidation of *in situ* ligninolysis mechanisms of the selective white-rot *FungusCeriporiopsis subvermispora*. *ACS Sustain. Chem. Eng.* **7**, 16757–16764 (2019).
52. H . Guo, D. M. Miles-Barrett, A. R. Neal, T. Zhang, C. Li, N. J. Westwood, Unravelling the enigma of ligninOX: Can the oxidation of lignin be controlled? *Chem. Sci.* **9**, 702–711 (2018).
53. G. van Erven, A. F. Kleijn, A. Patyshakuliyeva, M. Di Falco, A. Tsang, R. P. De Vries, W. J. H. Van Berkel, M. A. Kabel, Evidence for ligninolytic activity of the ascomycete fungus *Podospora anserina*. *Biotechnol. Biofuels* **13**, 75 (2020).
54. S. Constant, H. L. J. Wienk, A. E. Frissen, P. De Peinder, R. Boelens, D. S. Van Es, R. J. H. Grisel, B. M. Weckhuysen, W. J. J. Huijgen, R. J. A. Gosselink, P. C. A. Bruijnincx, New insights into the structure and composition of technical lignins: A comparative characterisation study. *Green Chem.* **18**, 2651–2665 (2016).
55. G. van Erven, N. Nayan, A. S. M. Sonnenberg, W. H. Hendriks, J. W. Cone, M. A. Kabel, Mechanistic insight in the selective delignification of wheat straw by three white-rot fungal species through quantitative 13 C-IS py-GC–MS and whole cell wall HSQC NMR. *Biotechnol. Biofuels* **11**, 262 (2018).
56. J. M. Buescher, M. R. Antoniewicz, L. G. Boros, S. C. Burgess, H. Brunengraber, C. B. Clish, R. J. DeBerardinis, O. Feron, C. Frezza, B. Ghesquiere, E. Gottlieb, K. Hiller, R. G. Jones, J. J. Kamphorst, R. G. Kibbey, A. C. Kimmelman, J. W. Locasale, S. Y. Lunt, O. D. K. Maddocks, C. Malloy, C. M. Metallo, E. J. Meuillet, J. Munger, K. Nöh, J. D. Rabinowitz, M. Ralser, U. Sauer, G. Stephanopoulos, J. St-Pierre, D. A. Tennant, C. Wittmann, M. G. Vander Heiden, A. Vazquez, K. Vousden, J. D. Young, N. Zamboni, S. M. Fendt, A roadmap for interpreting 13C metabolite labeling patterns from cells. Elsevier Ltd [Preprint] (2015). https://doi.org/10.1016/j.copbio.2015.02.003, 34, 189, 201.
